# 5-Aryl-1,3,4-oxadiazol-2-amines Decorated with Long Alkyl and Their Analogues: Synthesis, Acetyl- and Butyrylcholinesterase Inhibition and Docking Study

**DOI:** 10.3390/ph15040400

**Published:** 2022-03-25

**Authors:** Václav Pflégr, Šárka Štěpánková, Katarína Svrčková, Markéta Švarcová, Jarmila Vinšová, Martin Krátký

**Affiliations:** 1 Department of Organic and Bioorganic Chemistry, Faculty of Pharmacy in Hradec Králové, Charles University, Akademika Heyrovského 1203, 500 05 Hradec Králové, Czech Republic; pflegrv@faf.cuni.cz (V.P.); komloova.m@seznam.cz (M.Š.); vinsova@faf.cuni.cz (J.V.); 2 Department of Biological and Biochemical Sciences, Faculty of Chemical Technology, University of Pardubice, Studentská 573, 532 10 Pardubice, Czech Republic; sarka.stepankova@upce.cz (Š.Š.); katarina.svrckova@upce.cz (K.S.); 3 Department of Chemistry, Faculty of Science, J. E. Purkinje University, Pasteurova 3632/15, 400 96 Ústí nad Labem, Czech Republic

**Keywords:** 1,3,4-oxadiazole, 1,3,4-thiadiazole, acetylcholinesterase, butyrylcholinesterase, enzyme inhibition, molecular docking

## Abstract

2,5-Disubstituted 1,3,4-oxadiazoles are privileged versatile scaffolds in medicinal chemistry that have exhibited diverse biological activities. Acetyl- (AChE) and butyrylcholinesterase (BChE) inhibitors are used, e.g., to treat dementias and myasthenia gravis. 5-Aryl-1,3,4-oxadiazoles decorated with dodecyl linked via nitrogen, sulfur or directly to this heterocycle have been designed as potential inhibitors of AChE and BChE. They were prepared from commercially available or in-house prepared hydrazides by reaction with dodecyl isocyanate to form hydrazine-1-carboxamides **2** (yields 67–98%) followed by cyclization using *p*-toluenesulfonyl chloride and triethylamine in 41–100% yields. Thiadiazole isostere was also synthesized. The derivatives were screened for inhibition of AChE and BChE using Ellman’s spectrophotometric method. The compounds showed a moderate dual inhibition with IC_50_ values of 12.8–99.2 for AChE and from 53.1 µM for BChE. All the heterocycles were more efficient inhibitors of AChE. The most potent inhibitor, *N*-dodecyl-5-(pyridin-4-yl)-1,3,4-thiadiazol-2-amine **3t**, was subjected to advanced reversibility and type of inhibition evaluation. Structure–activity relationships were identified. Many oxadiazoles showed lower IC_50_ values against AChE than established drug rivastigmine. According to molecular docking, the compounds interact non-covalently with AChE and BChE and block entry into enzyme gorge and catalytic site, respectively.

## 1. Introduction

Acetylcholine (ACh) is a potent neurotransmitter in both the central nervous system and the peripheral nervous system, that plays an important role in performing the cognitive functions. Enzyme acetylcholinesterase (AChE), which cleaves choline esters, is a serine hydrolase found mainly at neuromuscular junctions and cholinergic brain synapses. The enzyme inactivation induced by various inhibitors leads to acetylcholine accumulation, hyperstimulation of nicotinic and muscarinic receptors, and disruption of neurotransmission [1]. Uncontrolled acetylcholine can lead to a massive disturbance in the cholinergic system, respiratory arrest, and death [2].

According to the mode of action, AChE inhibitors can be divided into two groups: irreversible and reversible. Reversible competitive or non-competitive inhibitors (donepezil, rivastigmine, galantamine) are the protagonists of pharmacotherapy of Alzheimer disease symptoms. Their therapeutic effect is based on maintaining the level of ACh by slowing down its hydrolysis rate. Reversible AChE inhibitors of various chemical structures have noteworthy pharmacological application in the treatment of neurological disorders such as myasthenia gravis, Lewy bodies and Parkinson’s disease dementia, and as well as prophylactics against nerve agent intoxication [3].

Butyrylcholinesterase (BChE) is a sister enzyme to AChE, which was discovered more than 80 years ago [4] and is found throughout the body [5]. BChE catalyzes the hydrolysis of numerous esters of choline such as succinylcholine, adipoyl choline, benzoyl choline, and neurotoxic peptides [6,7]. Although AChE is a primary enzyme for the hydrolysis of ACh, BChE is a putative backup enzyme for this reaction. Changes in the homeostasis of ACh and its destruction by AChE involve numerous impairments such as muscular dystrophy, motor neuron diseases such as amyotrophic lateral sclerosis [8], congenital myasthenias [9] and myasthenia gravis [10]. While the reason for the widespread presence of BChE in the body is not well understood [11], it can contribute to the inactivation of the neurotransmitter acetylcholine, degrading many neurotoxic organophosphate esters and natural poisons including solanidine, physostigmine, cocaine, and others [12]. Thus, it plays important pharmacological and toxicological roles and is thought to be involved in the pathological progression. Based on these facts, a dual inhibition strategy for both these enzymes has been proposed to increase the effectiveness of the treatment strategy and expand the indications [13].

The five-membered ring 1,3,4-oxadiazole, one of four isomers, is a thermally stable aromatic molecule that was first prepared by Ainsworth in 1965 by the thermolysis of ethyl (*E*)-*N*-formylformohydrazonate at atmospheric pressure [14]. Synthesis approaches are summarized in the review article of Patel and co-authors [15]. The replacement of two –CH= groups in furan by two nitrogens (–N=) reduces the aromaticity of the resulting oxadiazole ring to such an extent that the oxadiazole ring exhibits the character of conjugated diene [16]. They undergo several reactions such as photochemical, thermal, electrophilic, and nucleophilic substitutions [17]. Several molecules with this valued low-lipophilicity moiety plays significant role in medicinal chemistry due to their diverse biological activities. They are widely applied in the development of novel compounds with anticonvulsant, antidepressant, analgesic, anti-inflammatory, antiallergic, antipsychotic, antimicrobial, antimycobacterial, antitumor and antiviral activities [18,19,20,21]. 1,3,4-Oxadiazole heterocycles are bioisosteres of amides and esters, which can significantly contribute to increased pharmacological activity by participating in hydrogen bonding interactions with the receptors [22].

**Figure 1:**
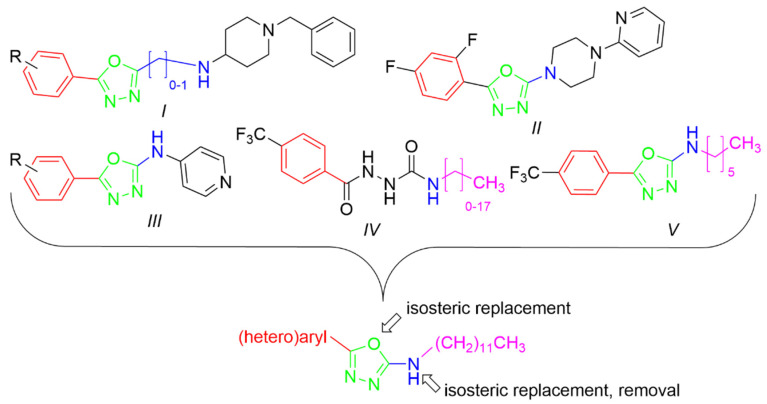
Structural fragments involved in the structure of targeted derivatives and their design: 1,3,4-oxadiazoles, long alkyl, aryl, and amine/amide linker.

In this work, we were focused primarily on 5-(hetero)aryl-1,3,4-oxadiazol-2-amines. Based on the literature, the amino group can be secondary, monosubstituted by (hetero)aryl or (cyclo)alkyl, or it can be incorporated into a heterocycle as a tertiary amine. For example, 5-aryl-1,3,4-oxadiazoles were tethered with donepezil analogue *N*-benzylpiperidine via (methyl)amino linker (Figure 1, *I*). The presence of oxadiazole resulted in the enhanced binding affinity toward the peripheral anionic site and was responsible for the extension of the *N*-benzylpiperidine moiety deeper into the catalytic anionic site, which yielded stronger dual inhibitors. Moreover, they showed antioxidant and neuroprotective properties and also inhibition of β-secretase-1 (BACE-1) and aggregation of amyloid β-peptide (Aβ). Additionally, 4-(pyridin-2-yl)piperazin-1-yl oxadiazole analogues (Figure 1, *II*) exhibited similar promising behavior together with adequate physicochemical properties [23,24]. 5-Aryl-*N*-(4-pyridyl)-1,3,4-oxadiazol-2-amines (Figure 1, *III*) were found to be dual inhibitors that also interfered with Aβ aggregation, oxidative stress, were blood–brain barrier permeable and exhibited in vivo activity in murine model [25]. The type of enzyme inhibition is not consistent, in that some derivatives were mixed [24], while some were non-competitive [25] inhibitors. Additionally, other authors have reported 2,5-disubstituted 1,3,4-oxadiazoles as AChE and/or BChE inhibitors, e.g., [26,27,28]. Thus, this scaffold belongs to multitargeting compounds that represent leading and the most perspective strategy to combat two or more pathogenetic pathways of neurodegeneration. From another point of view, oxadiazoles can be considered as cyclic analogues of 1,2-diacylhydrazines (Figure 1, *IV*) with also known activity against AChE and BChE [28,29]. (Hetero)arenes decorated by a longer alkyl (Figure 1, *V*) have been described as cholinesterases inhibitors as well; the length of this alkyl was crucial for bioactivity [28,30]. Additionally, 1,3,4-oxadiazol-2-thiols [31] and 1,3,4-thiadiazoles [32] have been investigated as potential cholinesterases inhibitors.

Considering the abovementioned facts, we designed, prepared, and evaluated *N*-dodecyl-5-substituted (hetero)aryl-1,3,4-oxadiazol-2-amines **3** and their isosteres **3s**–**3u** as potential inhibitors AChE and BChE.

## 2. Results and Discussion

### 2.1. Synthesis

**Figure 2:**
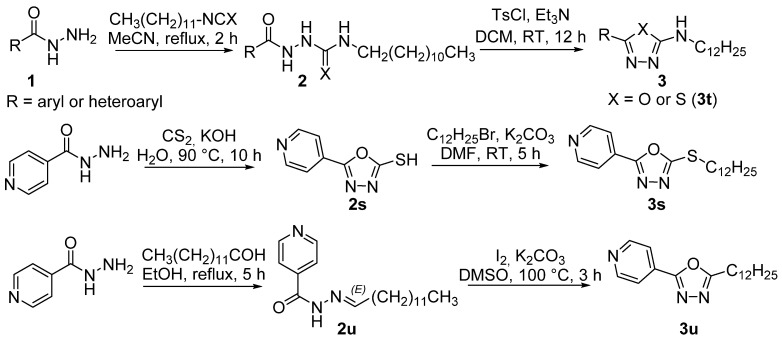
Synthesis of the targeted derivatives **3** (DCM: dichloromethane, DMF: *N*,*N*-dimethylformamide, DMSO: dimethylsulfoxide, TsCl: *p*-toluenesulfonyl chloride, RT: room temperature.

The synthetic scheme is illustrated in Figure 2. The synthesis of final oxadiazoles and their analogues **3a**–**3u** started from aryl hydrazides that were either commercially available or prepared in-house from corresponding acid via Fisher esterification (with MeOH in the presence of a catalytic amount of sulfuric acid) and subsequent hydrazinolysis of the methyl ester. Then, hydrazide was reacted with a mild excess of dodecyl iso(thio)cyanate in acetonitrile. Crude 2-aryloylhydrazine-1-carboxamides **2** were purified by recrystallization with moderate to excellent yields of 67–98%. The lowest yields were for 2-bromopyridine derivative **2q**, followed by 3,5-dinitrobenzohydrazide derivative **2k** and thioamide **2t** (for both 76%).

2-Aryl-*N*-dodecylhydrazine-1-carboxamides **2** (or carbothioamide **2t**) were treated with an excess of *p*-toluenesulfonyl chloride (3 eq.) and triethylamine (5 eq.) in dichloromethane (DCM). After purification, yields of 1,3,4-oxa(or thia)diazoles **3** were lower due to reduced conversion rate and/or less effective isolation (41–100%). In particular, oxadiazoles prepared from heterocyclic hydrazides were obtained in lower yields (derivatives of pyridazine **3r**, picolinohydrazide **3n**, 2-bromoisonicotinohydrazide **3q**, and pyrazine **3p**). The thiadiazole **3t** was isolated with satisfactorily yield of 68%.

Two analogues were prepared in a different way. Dodecylsulfanyl derivative **3s** was prepared from isonicotinoylhydrazide that was heated with an excess of carbon disulfide keeping a strong basic pH (73%). The 5-(pyridin-4-yl)-1,3,4-oxadiazol-2-thiol was treated with an over-stochiometric amount of 1-bromododecane as an alkylating agent and potassium carbonate in dimethylformamide. Thioether **3s** was obtained quantitatively. 5-Dodecyl derivative **3u** was synthesized from isoniazid by condensation reaction with tridecanal to form hydrazide-hydrazone **2u**. This intermediate was cyclized to oxadiazole using a mild excess of iodine and potassium carbonate (76%).

The prepared compounds were characterized by ^1^H and ^13^C NMR, IR spectra and melting point. The purity was confirmed by thin-layer chromatography and elemental analysis. In the ^1^H NMR spectra, the signals of heteroaryls and substituted phenyls are clearly visible, while signals of aliphatic hydrogens from the dodecyl chain partially overlap. Here, we can distinguish signals of C_1_, C_2_ and C_12_, remaining hydrogen signals are merged. By analogy, the signals of most aliphatic carbons in the ^13^C NMR spectra are close together. As expected, both hydrazide hydrogen signals in the spectra of **2** disappeared in the spectra of **3** due to cyclization. 

### 2.2. Inhibition of Acetyl- and Butyrylcholinesterase 

The heterocycles **3** were screened for their ability to interfere with the function of AChE from electric eel (*Ee*AChE) and BChE from equine serum (EqBChE) using Ellman’s method (Table 1). Their activities are expressed as the concentration producing 50% inhibition of enzymatic activity (IC_50_). In addition, we calculated selectivity indexes (SI) that quantify the selectivity for AChE as more inhibited cholinesterase. SI is the ratio of IC_50_ for BChE/IC_50_ for AChE. Clinically used AChE and BChE inhibitor rivastigmine was used as a comparator.

Most derivatives **3** caused dual inhibition of both cholinesterases (derivative **3s** was an exception). AChE was inhibited more consistently with lower IC_50_ values of 12.8–99.2 µM with thiadiazole **3t** being the most potent, followed by several 5-(4-substituted phenyl) derivatives: OMe (**3c**), Me (**3b**), F (**3g**), I (**3j**), Br (**3i**) and H (**3a**) with IC_50_ of 33.9–40.1 µM. More bulky substituents increased IC_50_ (*tert*-butyl, dimethylamino, 4-nitro). The conversion of thiadiazole to oxadiazole was detrimental (**3t** vs. **3l**; 3.9× lower inhibition), as was the removal of secondary amine or its replacement with sulfide (**3l** vs. **3s** and **3u**). Focusing on 5-heteroaryls, 4-pyrimidinyl showed the best inhibition (**3o**, 45.5 µM) followed by various positional isomers of pyridine (4-pyridyl **3l**, 2-pyridyl **3n** and 3-pyridyl **3m**; 50.2–64.2 µM). On the other hand, the pyrazine derivative **3p** was the least active in this subgroup.

In contrast, BChE enzyme was inhibited at higher concentrations, and their range was wider. The IC_50_ of the dodecylsulfanyl compound **3s** was higher than 500 µM. In fact, only the thiadiazole **3t** and 5-(*p*-tolyl)-oxadiazole **3b** were more potent inhibitors of BChE with IC_50_ of 53.05 and 89.97 µM, respectively. Remaining derivatives have IC_50_ ≥ 105 µM. Among the oxadiazoles derived from benzohydrazides **3a**–**3k**, the optimal activity was associated with 4-methyl (**3b**), while its replacement by hydrogen (**3a**), methoxy (**3c**) or *tert*-butyl (**3d**) led to a decrease in activity. Halogens and dimethylamino group were also unfavorable. Interestingly, only the 4-NO_2_ group with completely different properties led to a comparably active derivative. Of the various 5-heteroaryls, the best inhibition is associated with 4-pyridyl (**3l**), but its bromination (**3q**) and isosteric replacement by pyridazine (**3r**) is tolerated. The switch of oxygen to sulfur (**3l** vs. **3t**) boosted inhibitory potency 4.2×. On the other hand, replacement of exocyclic NH group with sulfur completely abolished the activity (**3l** vs. **3s**), whereas its removal resulted in only a slight decrease in inhibition (**3l** vs. **3u**).

We used the selectivity index values to describe the selectivity to particular ACh hydrolysing enzymes. All the derivatives were preferential inhibitors of AChE (SI within the range from 1.5 to 12.9). The most selective was iodinated derivative **3i**; on the other hand, 4-nitro compound **3e** was the most balanced inhibitor. Interestingly, an introduction of the second nitro group (**3k**) and increasing molecular weight of halogens (**3g** → **3j**) resulted in a higher selectivity to AChE. Among the derivatives with a heterocycle on carbon 5, the least selective are brominated pyridine **3q** and pyridazine **3r**. Positional isomers (**3m** and **3n**) of isoniazid-derived oxadiazole **3l** are more selective to AChE. The removal of a heteroatom at the position 2 of oxadiazole led to more balanced inhibition (**3l**, **3s**, and **3t** vs. **3u**).

Then, we tried to correlate activity with calculated lipophilicity (log *P_o/w_*; Table 1). However, there are no clear conclusive structure–activity relationships and differences in lipophilicity cannot fully explain differences in activity. For example, compounds with close log *P* values (**3s**–**3u**) showed different degrees of inhibition of both enzymes. The most potent AChE inhibitors were lowly lipophilic (**3t**, **3k**), moderately (**3c**) and also highly lipophilic (**3j**). Analogously, compounds with the same log *P* values (**3e**, **3l**–**3n**, and **3t**) inhibited BChE over a wide concentration range of 53–369 µM.

When comparing the heterocycles **3** with the drug rivastigmine, none of the new compounds had lower IC_50_ values for BChE than this clinically used dual AChE/BChE inhibitor. By contrast, many of them (**3a**–**3c**, **3g**–**3k**, **3l**, **3o**, and **3t**) showed higher in vitro activities expressed as IC_50_ (up to 4.4× for **3t**) against AChE.

#### 2.2.1. Reversibility of the Inhibition

Based on the promising in vitro inhibition of AChE by thiadiazole **3t**, we investigated the derivative to distinguish between reversible, pseudo-irreversible and irreversible inhibition on the basis of changes in enzyme activity over time in the presence of the inhibitor. In reversible inhibition, the activity of enzyme goes down immediately, but the inhibitory molecule is bound to the target for a short period of time, after which the inhibitor-enzyme complex dissociates, and enzyme activity is restored. In pseudo-irreversible inhibition, the inhibitor binds covalently to the enzyme, but the bond is broken down more slowly, delaying the return of enzyme activity to its initial state. In the case of irreversible inhibitors, the reaction between the enzyme and the inhibitor is not rapid because there is a time-dependent drop of activity. Irreversible inhibitors decrease enzyme activity gradually. The inhibitor permanently binds to the enzyme and prevents the enzyme from restoring its activity [33].

**Figure 3:**
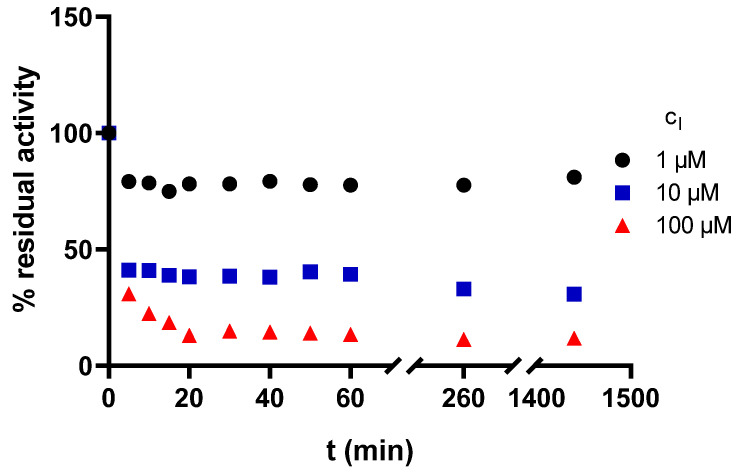
The dependence of enzyme residual activity on time (enzyme: AChE, compound **3t**).

Three different concentrations were chosen for **3t** according to its IC_50_ value for AChE. The first concentration was lower than IC_50_, the second was close to IC_50_ and the third was higher than IC_50_ (1, 10, and 100 µM, respectively). All obtained dependencies of A (expressed as residual activity on the Y-axis) versus time showed a very similar pattern (Figure 3). First, a rapid decrease in activity (for two lower concentrations, almost maximum inhibition is reached after 5 min), then a plateau phase followed by a recovery of enzyme activity over time.

In our opinion, we can conclude that the compound **3t** acts as reversible AChE inhibitor. 

#### 2.2.2. Type of Inhibition

We also valuated the type of inhibition of **3t** using AChE. In general, the reversible inhibitors can be classified as competitive, non-competitive, uncompetitive, or mixed type. The type of inhibition could be distinguished using the Lineweaver–Burk plot [34] and the corresponding comparison of two key kinetic parameters: maximum velocity (V_m_) and Michaelis constant (K_M_) of the inhibited and uninhibited reactions. Based on the changes of these parameters and intercept of the lines in Lineweaver–Burk plot, the type of inhibition is uncovered. 

**Figure 4:**
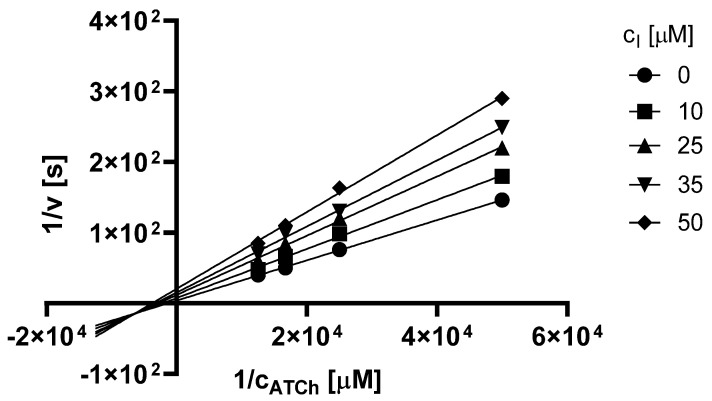
Lineweaver–Burk plot for **3t** and AChE inhibition (ATCh = acetylthiocholine).

The Lineweaver–Burk plot obtained for **3t** and AChE is shown in Figure 4. Based on the plot, it can be concluded that this heterocycle causes a mixed type of inhibition. The inhibition is associated with a change in both K_M_ and V_m_ and also their ratio is different compared to the uninhibited reaction. In the Lineweaver–Burk plot, the intersection of the lines is in quadrant III but not on axis.

### 2.3. Molecular Docking 

For better visualization of the possible orientations of prepared compounds in enzymes, molecular modelling study was performed. Based on the obtained data, the structure–activity relationships were estimated.

**Figure 5:**
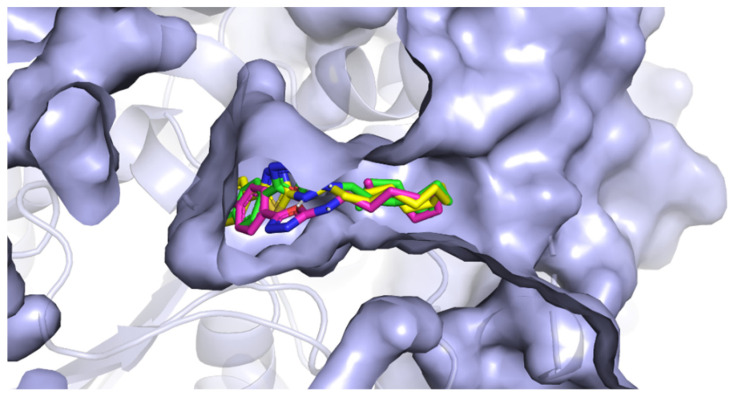
The position of **3b**, **3c**, and **3t** in the cavity of AChE.

**Figure 6:**
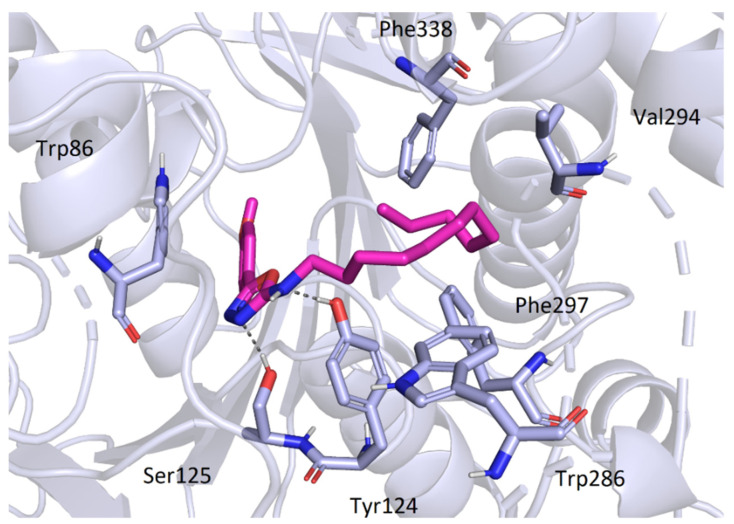
Binding mode of the compound **3c** in AChE.

The active site of AChE was studied extensively in the past and is now very well described. It is known to be at the bottom of a very narrow gorge penetrating deep under the surface of the enzyme. All the prepared most active ligands (**3b**, **3c**, **3t**) displayed similar orientation in the cavity of AChE (Figure 5) which corresponds with the results of in vitro testing, since the obtained activities were all in relatively small range. Variable 5-aryloxadiazole moiety was heading for the bottom of the gorge (though still quite far from the active site triad). Hydrophobic 2-dodecylamino moiety was convoluted, pointing out of the cavity, and blocking the entrance to the gorge. Several hydrophobic interactions with Trp286, Val294, Phe297, and Phe338 were observed. Interestingly, an important structural element seems to be the -NH- group at the dodecylamino moiety, which is stabilized at its position by hydrogen bonds (Asp74 or Tyr124). The replacement of the nitrogen with carbon or sulfur leads to decrease in the binding affinity (and also decrease in the in vitro inhibition data). The oxadiazole part of the molecules showed hydrogen bonding with Ser125. Aromatic/heteroaromatic moieties were located at the anionic site and displayed π-π stacking with Trp86 (Figure 6).

**Figure 7:**
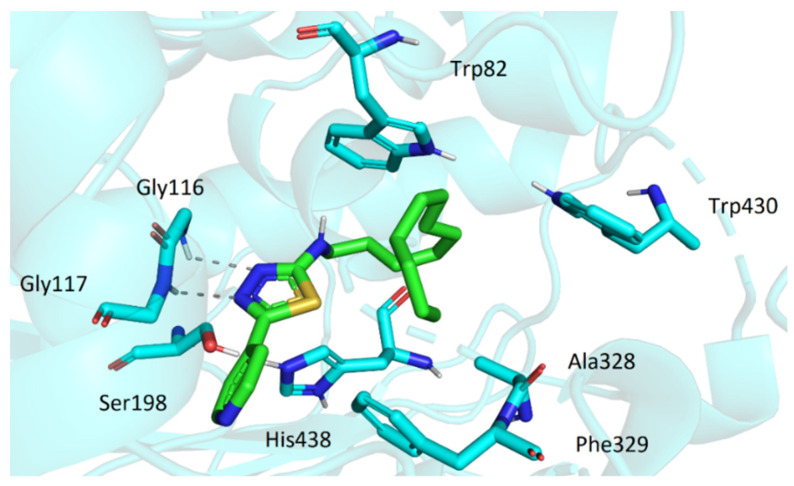
The top score docking pose of **3t** in active site of BChE.

**Figure 8:**
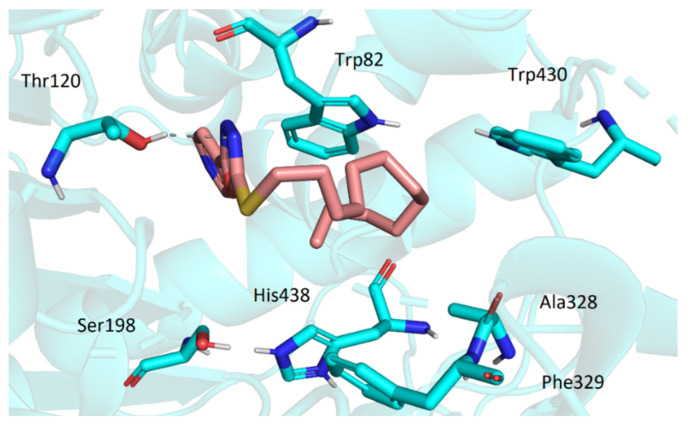
The conformation of **3s** in the active site of BChE.

Similarly to AChE, the active site of BChE is located at the bottom of the gorge. However, a considerable number of aromatic residues present in active center of AChE is replaced by smaller hydrophobic residues in BChE. This allows more spacious ligands to enter the cavity. The top score docking pose of **3t** (being the most potent inhibitor in the series) showed the molecule deep within the cavity in considerable proximity to active site Ser198 and His438 (Figure 7). The ligand was stabilized in its conformation by several interactions (namely, hydrogen bonds of oxadiazole moiety with Gly116 and Gly117 and hydrophobic interactions of dodecylamino moiety with Trp82, Ala328, Phe329, and Trp430). Most of the investigated compounds exhibited the same or quite similar conformation, apart from **3s**. Its dodecylamino moiety was placed in the same hydrophobic part of the cavity; however, the aryloxadiazole moiety was relatively far from Ser198 and His438 and showed only one interaction with Thr120 (Figure 8). This could stand behind its low ability to inhibit BChE.

## 3. Materials and Methods

### 3.1. Chemistry

#### 3.1.1. General

All chemicals for synthesis and analysis were purchased from Merck KGaA (Darmstadt, Germany), Penta Chemicals Unlimited (Prague, Czech Republic), Avantor (Stříbrná Skalice, Czech Republic), and Lach-Ner (Neratovice, Czech Republic) and were used as received. The structures of the prepared substances were confirmed by ^1^H NMR and ^13^C NMR spectroscopy analysis. NMR spectra were measured in dimethylsulfoxide (DMSO-*D_6_*) as a solvent at ambient temperature or with tempering the sample (**2b**–**2j**, **2q**, **3b**–**3k**, **3m**, **3o**–**3q**, and **3u**) at 60 °C by a JNM-ECZ 600R (600 MHz for ^1^H a 151 MHz for ^13^C; JEOL, Tokio, Japan) or a Varian VNMR S500 instrument (Varian Comp., Palo Alto, CA, USA). The chemical shifts δ are given in ppm and were referred indirectly to tetramethylsilane via residual signals of the solvent (2.50 for ^1^H and 39.51 for ^13^C spectra). The coupling constants (*J*) are reported in Hz. Infrared spectra were recorded by a Nicolet 6700 FT-IR spectrometer (Thermo Fisher Scientific, Waltham, MA, USA) in the range of 600–4000 cm^−1^, ATR (Ge) technique of measuring was used. Elemental analysis was performed on Vario MICRO Cube Element Analyzer (Elementar Analysensysteme, Hanau, Germany). Calculated as well as found values are given as percentages. Melting points were recorded using a Büchi B-545 apparatus (BÜCHI Labortechnik AG, Flawil, Switzerland) without corrections. Retention factors (*R_f_*) of all prepared compounds, as well as reaction progresses, were analyzed by thin layer chromatography (TLC); the plates were coated with 0.2 mm Merck 60 F254 silica gel (Merck Millipore, Darmstadt, Germany) and were visualized by UV irradiation (254 nm). Dichloromethane (DCM)/methanol (93:7 *v*/*v*) mixture was used as the eluent.

Lipophilicity was calculated in silico using a free web tool SwissADME (http://www.swissadme.ch/index.php, accessed on 16 March 2022). It is expressed as log *P*_o/w_ (MLOGP).

#### 3.1.2. Synthesis

##### Synthesis of 2-Aryloyl-*N*-Dodecylhydrazine-1-(thio)Carboxamides (**2a**–**2r**) and Other Precursors (**2s**–**2u**)

Appropriate aryl hydrazide (1.0 mmol) was suspended in 10 mL of acetonitrile and heated to initiate boiling of the mixture, then dodecyl isocyanate (or dodecyl isothiocyanate for precursor of **3t**; 1.05 of equivalents, 1.05 mmol) was added quickly in one portion. The reaction mixture was refluxed continuously for 2 h, then left to cool down at RT and stored for 1 h at −20 °C. Solid material was filtered off and recrystallized from methanol to provide pure hydrazides **2**. The progress of the reaction was monitored by TLC.

2-Benzoyl-*N*-dodecylhydrazine-1-carboxamide **2a**. White solid; yield 98%; mp 137–137 °C. IR (ATR): 608, 661, 689, 749, 902, 1026, 1065, 1246, 1275, 1465, 1489, 1540, 1563, 1662, 1682, 2850, 2917, 2954, 3296 cm^−1^. ^1^H NMR (600 MHz, DMSO-*D*_6_) δ 10.05 (1H, s, NH-CO-NH-CH_2_), 7.85 (2H, dd, *J* = 7.1, 1.7 Hz, H2, H6), 7.73 (1H, s, NH-CO-Ar), 7.53–7.49 (1H, m, H4), 7.43 (2H, t, *J* = 7.7 Hz, H3, H5), 6.40 (1H, t, *J* = 5.8 Hz, NH-CH_2_), 2.97 (2H, q, *J* = 6.6 Hz, NH-CH_2_), 1.34 (2H, p, *J* = 7.0 Hz, NH-CH_2_-CH_2_), 1.21–1.19 (18H, m, C^3^H_2_, C^4^H_2_, C^5^H_2_, C^6^H_2_, C^7^H_2_, C^8^H_2_, C^9^H_2_, C^10^H_2_, C^11^H_2_), 0.82 (3H, t, *J* = 7.0 Hz, CH_3_). ^13^C NMR (151 MHz, DMSO-*D*_6_) δ 166.82, 158.87, 133.32, 132.12, 128.79, 128.07, 31.84, 30.41, 29.61, 29.56, 29.38, 29.36, 29.34, 29.32, 29.26, 26.85, 22.63, 14.48. Elemental analysis for C_20_H_33_N_3_O_2_ (347.50); calculated: C, 69.13; H, 9.57; N, 12.09, found: C, 68.19; H, 9.44; N, 12.30. *R_f_*: 0.66.

*N*-Dodecyl-2-(4-methylbenzoyl)hydrazine-1-carboxamide **2b**. White solid; yield 95%; mp 121–122 °C. IR (ATR): 608, 661, 721, 746, 838, 911, 1191, 1252, 1265, 1338, 1469, 1495, 1542, 1583, 1613, 1642, 1665, 2849, 2917, 2954, 3294 cm^−1^. ^1^H NMR (600 MHz, DMSO-*D*_6_) δ 9.96 (1H, s, NH-CO-NH-CH_2_), 7.75 (2H, d, *J* = 8.2 Hz, H2, H6), 7.69 (1H, s, NH-CO-Ar), 7.24 (2H, d, *J* = 8.1 Hz, H3, H5), 6.37 (1H, t, *J* = 5.8 Hz, NH-CH_2_), 2.96 (2H, q, *J* = 6.6 Hz, NH-CH_2_), 2.32 (3H, s, Ph-CH_3_), 1.38–1.29 (2H, m, NH-CH_2_-CH_2_), 1.21–1.18 (18H, m, C^3^H_2_, C^4^H_2_, C^5^H_2_, C^6^H_2_, C^7^H_2_, C^8^H_2_, C^9^H_2_, C^10^H_2_, C^11^H_2_), 0.82 (3H, t, *J* = 6.8 Hz, CH_3_). ^13^C NMR (151 MHz, DMSO-*D*_6_) δ 166.73, 158.92, 142.05, 130.53, 129.31, 128.09, 31.84, 30.41, 29.61, 29.56, 29.42, 29.40, 29.38, 29.36, 29.26, 26.85, 22.63, 21.52, 14.48. Elemental analysis for C_21_H_35_N_3_O_2_ (361.53); calculated: C, 69.77; H, 9.76; N, 11.62, found: C, 69.89; H, 9.70; N, 11.73. *R_f_*: 0.60.

*N*-Dodecyl-2-(4-methoxybenzoyl)hydrazine-1-carboxamide **2c**. White solid; yield 95%; mp 158–159 °C. IR (ATR): 605, 726, 756, 841, 912, 1038, 1127, 1181, 1254, 1335, 1463, 1504, 1540, 1564, 1609, 1642, 2850, 2921, 2956, 3310 cm^−1^. ^1^H NMR (600 MHz, DMSO-*D*_6_) δ 9.90 (1H, s, NH-CO-NH-CH_2_), 7.83 (2H, d, *J* = 9.1 Hz, H2, H6), 7.66 (1H, s, NH-CO-Ar), 6.96 (2H, d, *J* = 8.9 Hz, H3, H5), 6.37 (1H, t, *J* = 5.9 Hz, NH-CH_2_), 3.77 (3H, s, OCH_3_), 2.96 (2H, q, *J* = 6.7 Hz, NH-CH_2_), 1.37–1.30 (2H, m, NH-CH_2_-CH_2_), 1.21–1.19 (18H, m, C^3^H_2_, C^4^H_2_, C^5^H_2_, C^6^H_2_, C^7^H_2_, C^8^H_2_, C^9^H_2_, C^10^H_2_, C^11^H_2_), 0.82 (3H, t, *J* = 6.9 Hz, CH_3_). ^13^C NMR (151 MHz, DMSO-*D*_6_) δ 166.36, 162.40, 159.00, 129.94, 125.49, 114.02, 55.90, 31.84, 30.41, 29.65, 29.63, 29.61, 29.59, 29.57, 29.38, 29.26, 26.85, 22.63, 14.48. Elemental analysis for C_21_H_35_N_3_O_3_ (377.53); calculated: C, 66.81; H, 9.34; N, 11.13, found: C, 66.85; H, 9.38; N, 11.14. *R_f_*: 0.51.

2-[4-(*tert*-Butyl)benzoyl]-*N*-dodecylhydrazine-1-carboxamide **2d**. White solid; yield 91%; mp 119–120 °C. IR (ATR): 621, 632, 718, 897, 1018, 1083, 1113, 1200, 1290, 1305, 1364, 1464, 1506, 1537, 1614, 1668, 2853, 2924, 2958, 3250 cm^−1^. ^1^H NMR (600 MHz, DMSO-*D*_6_) δ 9.98 (1H, s, NH-CO-NH-CH_2_), 7.79 (2H, d, *J* = 8.7 Hz, H2, H6), 7.71 (1H, s, NH-CO-Ar), 7.44 (2H, d, *J* = 8.7 Hz, H3, H5), 6.37 (1H, t, *J* = 5.8 Hz, NH-CH_2_), 2.96 (2H, q, *J* = 6.7 Hz, NH-CH_2_), 1.34 (2H, q, *J* = 7.0 Hz, NH-CH_2_-CH_2_), 1.26 (9H, s, C(CH_3_)_3_), 1.23–1.19 (18H, m, C^3^H_2_, C^4^H_2_, C^5^H_2_, C^6^H_2_, C^7^H_2_, C^8^H_2_, C^9^H_2_, C^10^H_2_, C^11^H_2_), 0.81 (3H, t, *J* = 7.0 Hz, CH_3_). ^13^C NMR (151 MHz, DMSO-*D*_6_) δ 166.68, 158.92, 154.96, 130.56, 127.94, 125.53, 35.17, 31.85, 31.45, 30.40, 29.68. 29.66, 29.64, 29.62, 29.57, 29.39, 29.27, 26.85, 22.64, 14.48. Elemental analysis for C_24_H_41_N_3_O_2_ (403.61); calculated: C, 71.42; H, 10.24; N, 10.41, found: C, 71.55; H, 10.29; N, 10.40. *R_f_*: 0.62.

*N*-Dodecyl-2-(4-nitrobenzoyl)hydrazine-1-carboxamide **2e**. White solid; yield 88%; mp 220–221 °C. IR (ATR): 625, 679, 713, 852, 868, 911, 1011, 1056, 1250, 1263, 1299, 1316, 1344, 1468, 1484, 1534, 1551, 1590, 1605, 1641, 1671, 2852, 2920, 3275 cm^−1^_._^1^H NMR (600 MHz, DMSO-*D*_6_) δ 10.26 (1H, s, NH-CO-NH-CH_2_), 8.27 (2H, d, *J* = 8.8 Hz, H3, H5), 8.07 (2H, d, *J* = 8.8 Hz, H2, H6), 7.76 (1H, s, NH-CO-Ar), 6.35 (1H, t, *J* = 6.1 Hz, NH-CH_2_), 3.00 (2H, q, *J* = 6.6 Hz, NH-CH_2_), 1.37 (2H, q, *J* = 7.0 Hz, NH-CH_2_-CH_2_), 1.23–1.20 (18H, m, C^3^H_2_, C^4^H_2_, C^5^H_2_, C^6^H_2_, C^7^H_2_, C^8^H_2_, C^9^H_2_, C^10^H_2_, C^11^H_2_), 0.82 (3H, t, *J* = 7.0 Hz, CH_3_). ^13^C NMR (151 MHz, DMSO-*D*_6_) δ 165.28, 158.52, 149.91, 139.22, 129.58, 123.91, 31.77, 30.33, 29.53, 29.50, 29.48, 29.46, 29.44, 29.30, 29.16, 26.83, 22.53, 14.33. Elemental analysis for C_20_H_32_N_4_O_4_ (392.50); calculated: C, 61.20; H, 8.22; N, 14.27, found: C, 61.29; H, 8.11; N, 14.36. *R_f_*: 0.51.

2-[4-(Dimethylamino)benzoyl]-*N*-dodecylhydrazine-1-carboxamide **2f**. White solid; yield 88%; mp 160–161 °C. IR (ATR): 609, 721, 763, 836, 950, 1068, 1173, 1211, 1265, 1288, 1340, 1375, 1468, 1515, 1540, 1556, 1607, 1637, 1685, 2850, 2918, 2952, 3292 cm^−1^. ^1^H NMR (600 MHz, DMSO-*D*_6_) δ 9.72 (1H, s, NH-CO-NH-CH_2_), 7.75 (2H, d, *J* = 9.0 Hz, H2, H6), 7.59 (1H, s, NH-CO-Ar), 6.70 (2H, d, *J* = 8.9 Hz, H3, H5), 6.33 (1H, t, *J* = 5.8 Hz, NH-CH_2_), 3.02–2.95 (8H, m, N-CH_3_, NH-CH_2_), 1.37 (2H, p, *J* = 6.9 Hz, NH-CH_2_-CH_2_), 1.28–1.19 (18H, m, C^3^H_2_, C^4^H_2_, C^5^H_2_, C^6^H_2_, C^7^H_2_, C^8^H_2_, C^9^H_2_, C^10^H_2_, C^11^H_2_), 0.85 (3H, t, *J* = 6.8 Hz, CH_3_). ^13^C NMR (151 MHz, DMSO-*D*_6_) δ 166.42, 158.81, 152.51, 129.08, 119.38, 110.85, 39.85, 31.48, 30.06, 29.26, 29.25, 29.24, 29.21, 29.20, 29.02, 28.90, 26.49, 22.27, 14.12. Elemental analysis for C_22_H_38_N_4_O_2_ (390.57); calculated: C, 67.66; H, 9.81; N, 14.35, found: C, 67.75; H, 9.90; N, 14.34. *R_f_*: 0.46.

*N*-Dodecyl-2-(4-fluorobenzoyl)hydrazine-1-carboxamide **2g**. White solid; yield 88%; mp 202–203 °C. IR (ATR): 610, 645, 726, 849, 914, 1011, 1164, 1232, 1262, 1339, 1466, 1502, 1544, 1569, 1605, 1638, 1661, 2851, 2921, 3301 cm^−1^. ^1^H NMR (600 MHz, DMSO-*D*_6_) δ 10.08 (1H, s, NH-CO-NH-CH_2_), 7.95–7.89 (2H, m, H2, H6), 7.74 (1H, s, NH-CO-Ar), 7.31–7.24 (2H, m, H3, H5), 6.43 (1H, t, *J* = 5.8 Hz, NH-CH_2_), 2.96 (2H, q, *J* = 6.6 Hz, NH-CH_2_), 1.35–1.32 (2H, m, NH-CH_2_-CH_2_), 1.21–1.19 (18H, m, C^3^H_2_, C^4^H_2_, C^5^H_2_, C^6^H_2_, C^7^H_2_, C^8^H_2_, C^9^H_2_, C^10^H_2_, C^11^H_2_), 0.81 (3H, t, *J* = 7.0 Hz, CH_3_). ^13^C NMR (151 MHz, DMSO-*D*_6_) δ 165.64 (d, *J* = 54.1 Hz), 163.81, 158.84, 130.77 (d, *J* = 8.9 Hz), 129.81 (d, *J* = 2.9 Hz), 115.76 (d, *J* = 21.8 Hz), 31.84, 30.40, 29.61, 29.58, 29.56, 29.38, 29.36, 29.34, 29.26, 26.85, 22.63, 14.47. Elemental analysis for C_20_H_32_FN_3_O_2_ (365.49); calculated: C, 65.72; H, 8.83; N, 11.50, found: C, 65.80; H, 8.92; N, 11.51. *R_f_*: 0.48.

2-(4-Chlorobenzoyl)-*N*-dodecylhydrazine-1-carboxamide **2h**. White solid; yield 91%; mp 215–216 °C. IR (ATR): 602, 680, 719, 754, 845, 911, 1011, 1092, 1252, 1266, 1339, 1470, 1485, 1587, 1599, 1639, 1664, 2850, 2918, 2954, 3299 cm^−1^. ^1^H NMR (600 MHz, DMSO-*D*_6_) δ 10.13 (1H, s, NH-CO-NH-CH_2_), 7.86 (2H, d, *J* = 8.2 Hz, H2, H6), 7.76 (1H, s, NH-CO-Ar), 7.52 (2H, d, *J* = 8.3 Hz, H3, H5), 6.44 (1H, t, *J* = 5.8 Hz, NH-CH_2_), 2.96 (2H, q, *J* = 6.6 Hz, NH-CH_2_), 1.36–1.32 (2H, m, NH-CH_2_-CH_2_), 1.21–1.18 (18H, m, C^3^H_2_, C^4^H_2_, C^5^H_2_, C^6^H_2_, C^7^H_2_, C^8^H_2_, C^9^H_2_, C^10^H_2_, C^11^H_2_), 0.81 (3H, t, *J* = 6.9 Hz, CH_3_). ^13^C NMR (151 MHz, DMSO-*D*_6_) δ 165.85, 158.77, 136.98, 132.11, 130.01, 128.92, 31.84, 30.39, 29.67, 29.65, 29.63, 29.61, 29.56, 29.37, 29.26, 26.84, 22.63, 14.48. Elemental analysis for C_20_H_32_ClN_3_O_2_ (381.95); calculated: C, 62.89; H, 8.45; N, 11.00, found: C, 62.81; H, 8.56; N, 11.02. *R_f_*: 0.48.

2-(4-Bromobenzoyl)-*N*-dodecylhydrazine-1-carboxamide **2i**. White solid; yield 90%; mp 214–215 °C. IR (ATR): 607, 681, 719, 752, 845, 855, 911, 1015, 1033, 1254, 1260, 1266, 1339, 1474, 1499, 1587, 1599, 1639, 1664, 2854, 2928, 2954, 3250 cm^−1^. ^1^H NMR (600 MHz, DMSO-*D*_6_) δ 10.19 (1H, s, NH-CO-NH-CH_2_), 7.85 (2H, d, *J* = 8.3 Hz, H2, H6), 7.79 (1H, s, NH-CO-Ar), 7.70 (2H, d, *J* = 8.3 Hz, H3, H5), 6.46 (1H, t, *J* = 5.9 Hz, NH-CH_2_), 2.95 (2H, q, *J* = 6.6 Hz, NH-CH_2_), 1.37–1.33 (2H, m, NH-CH_2_-CH_2_), 1.22–1.18 (18H, m, C^3^H_2_, C^4^H_2_, C^5^H_2_, C^6^H_2_, C^7^H_2_, C^8^H_2_, C^9^H_2_, C^10^H_2_, C^11^H_2_), 0.82 (3H, t, *J* = 6.9 Hz, CH_3_). ^13^C NMR (151 MHz, DMSO-*D*_6_) δ 165.62, 158.40, 132.10, 131.49, 129.83, 125.54, 31.48, 30.03, 29.27, 29.25, 29.23, 29.21, 29.17, 29.02, 28.90, 26.48, 22.28, 14.13. Elemental analysis for C_20_H_32_BrN_3_O_2_ (426.40); calculated: C, 56.34; H, 7.56; N, 9.85, found: C, 56.42; H, 7.66; N, 9.79. *R_f_*: 0.51.

*N*-Dodecyl-2-(4-iodobenzoyl)hydrazine-1-carboxamide **2j**. White solid; yield 94%; mp 216–217 °C. IR (ATR): 623, 635, 718, 750, 840, 908, 1005, 1066, 1252, 1268, 1339, 1470, 1479, 1507, 1542, 1590, 1636, 1663, 2850, 2917, 2951, 3303 cm^−1^. ^1^H NMR (600 MHz, DMSO-*D*_6_) δ 10.11 (1H, s, NH-CO-NH-CH_2_), 7.84–7.82 (2H, m, H3, H5), 7.74 (1H, s, NH-CO-Ar), 7.63–7.61 (2H, m, H2, H6), 6.42 (1H, t, *J* = 5.8 Hz, NH-CH_2_), 2.96 (q, *J* = 6.8 Hz, NH-CH_2_), 1.36–1.31 (2H, m, NH-CH_2_-CH_2_), 1.21–1.19 (18H, m, C^3^H_2_, C^4^H_2_, C^5^H_2_, C^6^H_2_, C^7^H_2_, C^8^H_2_, C^9^H_2_, C^10^H_2_, C^11^H_2_), 0.81 (3H, t, *J* = 7.0 Hz, CH_3_). ^13^C NMR (151 MHz, DMSO-*D*_6_) δ 166.25, 158.76, 137.71, 132.78, 130.01, 99.82, 31.84, 30.38, 29.65, 29.63, 29.61, 29.59, 29.56, 29.37, 29.26, 26.84, 22.64, 14.49. Elemental analysis for C_20_H_32_IN_3_O_2_ (473.40); calculated: C, 50.74; H, 6.81; N, 8.88, found: C, 50.86; H, 6.90; N, 8.95. *R_f_*: 0.51.

2-(3,5-Dinitrobenzoyl)-*N*-dodecylhydrazine-1-carboxamide **2k**. Greyish solid, yield 76%; mp 157–159 °C. IR (ATR): 717, 732, 920, 1084, 1241, 1350, 1466, 1476, 1538, 1569, 1615, 1653, 2848, 2924, 3099, 3216, 3357 cm^−1^. ^1^H NMR (600 MHz, DMSO-*D*_6_) *δ* 10.81 (1H, s, NH-CO-NH-CH_2_), 9.06 (2H, d, *J* = 2.0 Hz, H2, H6), 8.99 (1H, t, *J* = 2.1 Hz, H4), 8.04 (1H, s, NH-CO-Ar), 6.67 (1H, s, NH-CH_2_), 3.02 (2H, q, *J* = 6.6 Hz, NH-CH_2_), 1.41–1.36 (2H, m, NH-CH_2-_CH_2_), 1.27–1.21 (18H, m, C^3^H_2_, C^4^H_2_, C^5^H_2_, C^6^H_2_, C^7^H_2_, C^8^H_2_, C^9^H_2_, C^10^H_2_, C^11^H_2_), 0.85 (3H, t, *J* = 6.9 Hz, CH_3_). ^13^C NMR (151 MHz, DMSO-*D*_6_) *δ* 162.70, 158.10, 148.31, 135.70, 128.01, 121.41, 39.20, 31.49, 30.06, 29.27, 29.25, 29.22, 29.20, 29.03, 28.91, 26.49, 22.29, 14.14. Elemental analysis for C_20_H_31_N_5_O_6_ (437.50); calculated C, 54.91; H, 7.14; N, 16.01, found: C, 55.01; H, 7.21; N, 16.00. *R_f_*: 0.50.

*N*-Dodecyl-2-isonicotinoylhydrazine-1-carboxamide **2l**. The synthesis and characterization of the compound was published previously by our group [21].

*N*-Dodecyl-2-nicotinoylhydrazine-1-carboxamide **2m**. White solid; yield 95%; mp 143–144 °C. IR (ATR): 632, 721, 826, 903, 1028, 1124, 1194, 1253, 1266, 1317, 1379, 1470, 1525, 1589, 1638, 1695, 2848, 2916, 2979, 3197, 3311 cm^−1^. ^1^H NMR (600 MHz, DMSO-*D*_6_) δ 10.25 (1H, s, NH-CO-NH-CH_2_), 9.00 (1H, dd, *J* = 2.3, 0.9 Hz, H2), 8.69 (1H, dd, *J* = 4.8, 1.7 Hz, H4), 8.21–8.15 (1H, m, H6), 7.82 (1H, s, NH-CO-Ar), 7.48 (1H, ddd, *J* = 7.9, 4.8, 0.9 Hz, H5), 6.50 (t, *J* = 5.9 Hz, NH-CH_2_), 3.00–2.94 (2H, m, NH-CH_2_), 1.37–1.32 (2H, m, NH-CH_2_-CH_2_), 1.21–1.18 (18H, m, C^3^H_2_, C^4^H_2_, C^5^H_2_, C^6^H_2_, C^7^H_2_, C^8^H_2_, C^9^H_2_, C^10^H_2_, C^11^H_2_), 0.81 (3H, t, *J* = 6.9 Hz, CH_3_). ^13^C NMR (151 MHz, DMSO-*D*_6_) δ 165.48, 158.70, 152.74, 149.17, 135.79, 128.98, 123.97, 31.84, 30.39, 29.61, 29.59, 29.57, 29.56, 29.55, 29.37, 29.25, 26.85, 22.63, 14.48. Elemental analysis for C_19_H_32_N_4_O_2_ (348.49); calculated: C, 65.48; H, 9.26; N, 16.08, found: C, 65.42; H, 9.15; N, 16.01. *R_f_*: 0.24.

*N*-Dodecyl-2-picolinoylhydrazine-1-carboxamide **2n**. White solid; yield 89%; mp 101–103 °C. IR (ATR): 613, 727, 747, 823, 1017, 1038, 1066, 1240, 1271, 1435, 1465, 1481, 1497, 1565, 1573, 1634, 1715, 2849, 2926, 2956, 3316, 3377 cm^−1^. ^1^H NMR (500 MHz, DMSO-*D*_6_) δ 10.13 (1H, s, NH-CO-NH-CH_2_), 8.65 (1H, dd, *J* = 4.6, 1.4 Hz, H6), 8.03–7.97 (2H, m, H3, H4), 7.85 (1H, s, NH-CO-Ar), 7.62 (1H, ddd, *J* = 6.9, 4.7, 2.5 Hz, H5), 6.37 (1H, t, *J* = 5.7 Hz, NH-CH_2_), 2.99 (2H, q, *J* = 6.6 Hz, NH-CH_2_), 1.37 (2H, p, *J* = 6.9 Hz, NH-CH_2_-CH_2_), 1.28–1.20 (18H, m, C^3^H_2_, C^4^H_2_, C^5^H_2_, C^6^H_2_, C^7^H_2_, C^8^H_2_, C^9^H_2_, C^10^H_2_, C^11^H_2_), 0.84 (3H, t, *J* = 6.8 Hz, CH_3_). ^13^C NMR (151 MHz, DMSO-*D*_6_) δ 163.71, 158.08, 149.65, 148.67, 137.84, 126.95, 122.41, 39.40, 31.47, 29.99, 29.24, 29.22, 29.21, 29.19, 29.00, 28.89, 26.48, 22.26, 14.11. Elemental analysis for C_19_H_32_N_4_O_2_ (348.49); calculated: C, 65.48; H, 9.26; N, 16.08, found: C, 65.59; H, 9.19; N, 16.19. *R_f_*: 0.49.

*N*-Dodecyl-2-(pyrimidine-4-carbonyl)hydrazine-1-carboxamide **2o**. White solid, yield 89%; mp 133–134 °C. IR (ATR): 605, 639, 664, 765, 870, 997, 1096, 1157, 1231, 1289, 1342, 1389, 1466, 1506, 1556, 1582, 1659, 1696, 2852, 2922, 3090, 2922, 3090, 3214, 3292 cm^−1^. ^1^H NMR (600 MHz, DMSO-*D*_6_) δ 10.46 (1H, s, NH-CO-NH-CH_2_), 9.33 (1H, d, *J* = 1.5 Hz, H2), 9.07 (1H, d, *J* = 5.0 Hz, H5), 8.00 (1H, dd, *J* = 5.0, 1.4 Hz, H6), 7.95 (1H, s, NH-CO-Ar), 6.43 (1H, t, *J* = 5.7 Hz, NH-CH_2_), 2.99 (2H, q, *J* = 6.8 Hz, NH-CH_2_), 1.37 (2H, p, *J* = 6.9 Hz, NH-CH_2_-CH_2_), 1.29–1.20 (18H, m, C^3^H_2_, C^4^H_2_, C^5^H_2_, C^6^H_2_, C^7^H_2_, C^8^H_2_, C^9^H_2_, C^10^H_2_, C^11^H_2_), 0.84 (3H, t, *J* = 6.8 Hz, CH_3_). ^13^C NMR (151 MHz, DMSO-*D*_6_) δ 162.63, 159.69, 158.06, 157.82, 156.37, 119.01, 39.40, 31.46, 29.97, 29.23, 29.22, 29.19, 29.00, 28.88, 26.48, 22.26, 14.12. Elemental analysis for C_18_H_31_N_5_O_2_ (349.48); calculated C, 61.86; H, 8.94; N, 20.04, found: C, 61.95; H, 9.01; N, 20.15. *R_f_*: 0.32.

*N*-Dodecyl-2-(pyrazine-2-carbonyl)hydrazine-1-carboxamide **2p**. White solid, yield 79%; mp 128–132 °C. IR (ATR): 632, 725, 760, 915, 1021, 1072, 1117, 1170, 1224, 1263, 1268, 1408, 1466, 1490, 1567, 1644, 1698, 1717, 2849, 2923, 2955, 3310 cm^−1^. ^1^H NMR (600 MHz, DMSO-*D*_6_) δ 10.35 (1H, s, NH-CO-NH-CH_2_), 9.16 (1H, d, *J* = 1.5 Hz, H3), 8.88 (1H, d, *J* = 2.5 Hz, H6), 8.74 (1H, dd, *J* = 2.4, 1.5 Hz, H5), 7.91 (1H, s, NH-CO-Ar), 6.42 (1H, t, *J* = 5.7 Hz, NH-CH_2_), 3.00 (2H, q, *J* = 6.8 Hz, NH-CH_2_), 1.37 (2H, p, *J* = 6.9 Hz, NH-CH_2_-CH_2_), 1.29–1.20 (18H, m, C^3^H_2_, C^4^H_2_, C^5^H_2_, C^6^H_2_, C^7^H_2_, C^8^H_2_, C^9^H_2_, C^10^H_2_, C^11^H_2_), 0.85 (3H, t, *J* = 6.9 Hz, CH_3_). ^13^C NMR (151 MHz, DMSO-*D*_6_) δ 162.98, 157.99, 147.84, 144.81, 143.86, 143.61, 39.41, 31.48, 30.00, 29.27, 29.25, 29.24, 29.20, 29.01, 28.90, 26.49, 22.27, 14.11. Elemental analysis for C_18_H_31_N_5_O_2_ (349.48); calculated C, 61.86; H, 8.94; N, 20.04, found: C, 62.00; H, 9.14; N, 20.07. *R_f_*: 0.46.

2-(2-Bromoisonicotinoyl)-*N*-dodecylhydrazine-1-carboxamide **2q**. White solid; yield 67%; mp 169–171 °C. IR (ATR): 634, 725, 739, 929, 1078, 1245, 1261, 1337, 1370, 1462, 1539, 1567, 1650, 2850, 2923, 2953, 3040, 3281 cm^−1^. ^1^H NMR (600 MHz, DMSO-*D*_6_) δ 10.46 (1H, s, NH-CO-NH-CH_2_), 8.56 (1H, d, *J* = 5.1 Hz, H6), 8.03 (1H, d, *J* = 1.4 Hz, H3), 7.94 (1H, s, NH-CO-Ar), 7.81 (1H, dd, *J* = 5.1, 1.4 Hz, H5), 6.58 (1H, t, *J* = 5.9 Hz, NH-CH_2_), 3.00 (2H, q, *J* = 6.6 Hz, NH-CH_2_), 1.36 (2H, p, *J* = 6.9 Hz, NH-CH_2_-CH_2_), 1.28–1.19 (18H, m, C^3^H_2_, C^4^H_2_, C^5^H_2_, C^6^H_2_, C^7^H_2_, C^8^H_2_, C^9^H_2_, C^10^H_2_, C^11^H_2_), 0.84 (3H, t, *J* = 6.8 Hz, CH_3_). ^13^C NMR (151 MHz, DMSO-*D*_6_) δ 163.48, 158.06, 151.37, 143.17, 141.92, 126.05, 121.50, 39.36, 30.00, 29.31, 29.24, 29.20, 29.19, 19.11, 29.00, 28.89, 26.46, 22.27, 14.12. Elemental analysis for C_19_H_31_BrN_4_O_2_ (427.39); calculated: C, 53.40; H, 7.31; N, 13.11, found: C, 53.51; H, 7.22; N, 13.18. *R_f_*: 0.33.

*N*-Dodecyl-2-(pyridazine-4-carbonyl)hydrazine-1-carboxamide **2r**. White solid; yield 91%; mp 165–166 °C. IR (ATR): 644, 675, 716, 724, 753, 895, 979, 1052, 1247, 1265, 1288, 1351, 1464, 1481, 1545, 1569, 1620, 1662, 2849, 2922, 2954, 3080, 3177, 3349 cm^−1^. ^1^H NMR (600 MHz, DMSO-*D*_6_) δ 10.60 (1H, s, NH-CO-NH-CH_2_), 9.56 (1H, dd, *J* = 2.3, 1.3 Hz, H3), 9.45 (1H, dd, *J* = 5.2, 1.3 Hz, H6), 8.02 (1H, dd, *J* = 5.3, 2.3 Hz, H5), 7.98 (1H, s, NH-CO-Ar), 6.60 (1H, t, *J* = 5.7 Hz, NH-CH_2_), 3.01 (2H, q, *J* = 6.6 Hz, NH-CH_2_), 1.37 (2H, p, *J* = 6.9 Hz, NH-CH_2_-CH_2_), 1.28–1.20 (18H, m, C^3^H_2_, C^4^H_2_, C^5^H_2_, C^6^H_2_, C^7^H_2_, C^8^H_2_, C^9^H_2_, C^10^H_2_, C^11^H_2_), 0.85 (3H, t, *J* = 6.8 Hz, CH_3_). ^13^C NMR (151 MHz, DMSO-*D*_6_) δ 163.44, 157.99, 152.29, 148.91, 130.31, 124.53, 39.87, 31.45, 29.98, 29.94, 29.21, 29.19, 29.17, 28.97, 28.86, 26.45, 22.24, 14.02. Elemental analysis for C_18_H_31_N_5_O_2_ (349.48); calculated: C, 61.86; H, 8.94; N, 20.04, found: C, 61.90; H, 9.02; N, 20.14. *R_f_*: 0.13.

Isoniazid (10 mmol, 1.37 g) was dissolved in 50 mL of 5% aqueous solution of potassium hydroxide, then carbon disulfide (1.5 eq., 15 mmol, 1.14 g) was added in one portion. Reaction mixture was heated to 90 °C for 10 h. The mixture was cooled to room temperature and acidified to pH = 6 with 1M HCl. Residue precipitate was filtered off, washed with 10 mL of cold water and recrystallized from ethanol, gave pure 5-(pyridin-4-yl)-1,3,4-oxadiazol-2-thiol **2s** [35].

5-(Pyridin-4-yl)-1,3,4-oxadiazol-2-thiol **2s** [35]. Yellow solid, yield 73%; mp 264–266 °C. IR (ATR): 693, 722, 734, 828, 887, 1010, 1051, 1076, 1147, 1218, 1236, 1311, 1331, 1368, 1421, 1496, 1553, 1597, 1846, 2385 cm^−1^. ^1^H NMR (600 MHz, DMSO-*D*_6_) δ 8.82–8.79 (2H, m, H2, H6), 7.82–7.79 (2H, m, H3, H5). ^13^C NMR (151 MHz, DMSO-*D*_6_) δ 178.02, 158.94, 151.02, 129.95, 119.79. Elemental analysis for C_7_H_5_N_3_OS (179.20); calculated C, 46.92; H, 2.81; N, 23.45, found: C, 47.02; H, 2.95; N, 23.51. *R_f_*: 0.46.

*N*-Dodecyl-2-isonicotinoylhydrazine-1-carbothioamide **2t**. White solid, yield 76%; mp 199–201 °C. IR (ATR): 656, 692, 720, 752, 850, 908, 999, 1062, 1115, 1229, 1249, 1269, 1360, 1404, 1472, 1481, 1502, 1530, 1556, 1653, 2852, 2923, 3136, 3287 cm^−1^. ^1^H NMR (600 MHz, DMSO-*D*_6_) δ 10.59 (1H, s, NH-CS-NH-CH_2_), 9.32 (1H, s, NH-CS-Ar), 8.77–8.74 (2H, m, H2, H6), 8.14 (1H, t, *J* = 5.7 Hz, NH-CH_2_), 7.82–7.79 (2H, m, H3, H5), 3.41 (2H, q, *J* = 6.8 Hz, NH-CH_2_), 1.47 (2H, p, *J* = 7.0 Hz, NH-CH_2_-CH_2_), 1.29–1.19 (18H, m, C^3^H_2_, C^4^H_2_, C^5^H_2_, C^6^H_2_, C^7^H_2_, C^8^H_2_, C^9^H_2_, C^10^H_2_, C^11^H_2_), 0.85 (3H, t, *J* = 6.8 Hz, CH_3_). ^13^C NMR (151 MHz, DMSO-*D*_6_) δ 201.01, 164.58, 150.32, 139.78, 121.82, 43.91, 31.47, 29.24, 29.19, 29.17, 28.99, 28.89, 28.88, 28.86, 26.42, 22.27, 14.12. Elemental analysis for C_19_H_32_N_4_OS (364.55); calculated C, 62.60; H, 8.85; N, 15.37, found: C, 62.69; H, 8.92; N, 15.48. *R_f_*: 0.32.

Tridecanal (1 mmol, 198 mg) was dissolved in 20 mL of ethanol, 1 eq. of isoniazid (1 mmol, 137 mg) was added to the solution and reaction mixture was heated to reflux for 5 h. The mixture was reduced the volume in a rotary evaporator, and then stored in the freezer to crystalize (−20 °C). The solid product was filtered off and washed with methanol to provide pure hydrazone **2u** [36].

(*E*)-*N*′-Tridecylideneisonicotinohydrazide **2u** [37]. White solid, yield 90%; mp 90–92 °C. IR (ATR): 655, 670, 728, 754, 846, 877, 1043, 1295, 1367, 1412, 1460, 1548, 1598, 1624, 1655, 2848, 2923, 2955, 3070, 3257 cm^−1^. ^1^H NMR (600 MHz, DMSO-*D*_6_) δ 11.62 (1H, s, NH), 8.75–8.73 (2H, m, H2, H6), 7.76–7.73 (3H, m, H3, H5, N=CH), 2.26 (2H, td, *J* = 7.4, 5.5 Hz, =CH-CH_2_), 1.47 (2H, p, *J* = 7.1 Hz, =CH-CH_2_-CH_2_), 1.32–1.19 (18H, m, C^4^H_2_, C^5^H_2_, C^6^H_2_, C^7^H_2_, C^8^H_2_, C^9^H_2_, C^10^H_2_, C^11^H_2_, C^12^H_2_), 0.84 (3H, t, *J* = 6.9 Hz, CH_3_). ^13^C NMR (151 MHz, DMSO-*D*_6_) δ 161.36, 153.98, 150.42, 140.82, 121.63, 32.18, 31.48, 29.24, 29.21, 29.14, 28.99, 28.90, 28.83, 26.12, 22.28, 14.12. Elemental analysis for C_19_H_31_N_3_O (317.48); calculated C, 71.88; H, 9.84; N, 13.24, found: C, 71.95; H, 9.99; N, 13.15. *R_f_*: 0.31.

##### Procedure for Synthesis of 2-Aryloyl-N-Dodecyl-1,3,4-Oxadiazol-2-Amines (**3a**–**3r**) and Their Analogues (**3s**–**3u**)

An amount of 0.5 mmol of the appropriate 2-aryl-*N*-dodecylhydrazine-1-carboxamide (or *N*-dodecyl-2-isonicotinoylhydrazine-1-carbothioamide) **2** was suspended in 30 mL of DCM. Thereafter, 1.5 mmol (3 eq., 286 mg) of *p*-toluenesulfonyl chloride was added to the solution. After a complete dissolution of *p*-toluenesulfonyl chloride, 2.5 mmol of triethylamine (5 eq., 348 µL) was added dropwise. The mixture was allowed to react at room temperature for 12 h. The reaction mixture was evaporated to dryness on a rotary evaporator and transferred to a separatory funnel with 30 mL of ethyl acetate (EtOAc) and 30 mL of demineralized water. The organic phase was washed with 30 mL of a saturated NaHCO_3_ solution followed by 30 mL of a saturated brine. The organic phase was dried over anhydrous sodium sulfate for 45 min, then the desiccant was separated, and the mixture was evaporated to dryness. The crude product was overlaid with dry DCM and stored at −20 °C overnight. The solid product was filtered off and washed twice with 5 mL of ice-cold DCM. The progress of the reaction was monitored by TLC using a mixture of DCM with MeOH (93:7 *v*/*v*) as a mobile phase.

*N*-Dodecyl-5-phenyl-1,3,4-oxadiazol-2-amine **3a**. White solid, yield 100%; mp 114–115 °C. IR (ATR): 631, 635, 722, 750, 829, 833, 838, 1036, 1050, 1111, 1184, 1270, 1301, 1400, 1484, 1505, 1588, 1674, 2851, 2924, 3240 cm^−1^. ^1^H NMR (600 MHz, DMSO-*D*_6_) δ 7.87–7.67 (2H, m, H2, H6), 7.55–7.38 (3H, m, H3, H5, NH), 3.22 (2H, q, *J* = 6.6 Hz, NH-CH_2_), 1.56 (2H, p, *J* = 7.2 Hz, NH-CH_2_-CH_2_), 1.29–1.20 (18H, m, C^3^H_2_, C^4^H_2_, C^5^H_2_, C^6^H_2_, C^7^H_2_, C^8^H_2_, C^9^H_2_, C^10^H_2_, C^11^H_2_), 0.82 (3H, t, *J* = 6.9 Hz, CH_3_). ^13^C NMR (151 MHz, DMSO-*D*_6_) δ 164.35, 158.05, 130.77, 129.62, 125.65, 125.13, 43.30, 31.74, 29.48, 29.46, 29.44, 29.41, 29.39, 29.14, 29.11, 26.71, 22.48, 14.26. Elemental analysis for C_20_H_31_N_3_O (329.49); calculated C, 72.91; H, 9.48; N, 12.75, found: C, 73.00; H, 9.59; N, 12.64. *R_f_*: 0.77.

*N*-Dodecyl-5-(*p*-tolyl)-1,3,4-oxadiazol-2-amine **3b**. White solid, yield 91%; mp 121–122 °C. IR (ATR): 630, 724, 741, 829, 838, 1030, 1052, 1113, 1173, 1266, 1300, 1441, 1469, 1506, 1587, 1614, 2850, 2920, 3268 cm^−1^. ^1^H NMR (600 MHz, DMSO-*D*_6_) δ 7.66 (2H, d, *J* = 7.8 Hz, H2, H6), 7.41–7.39 (1H, m, NH), 7.29 (2H, d, *J* = 7.9 Hz, H3, H5), 3.20 (2H, td, *J* = 7.0, 5.7 Hz, NH-CH_2_), 2.33 (3H, s, Ph-CH_3_), 1.55 (2H, p, *J* = 7.1 Hz, NH-CH_2_-CH_2_), 1.24–1.17 (18H, m, C^3^H_2_, C^4^H_2_, C^5^H_2_, C^6^H_2_, C^7^H_2_, C^8^H_2_, C^9^H_2_, C^10^H_2_, C^11^H_2_), 0.82 (3H, t, *J* = 7.0 Hz, CH_3_). ^13^C NMR (151 MHz, DMSO-*D*_6_) δ 164.17, 158.16, 140.72, 130.19, 125.65, 122.41, 43.28, 31.74, 29.47, 29.44, 29.42, 29.40, 29.38, 29.13, 29.11, 26.70, 22.48, 21.45, 14.27. Elemental analysis for C_21_H_33_N_3_O (343.52); calculated C, 73.43; H, 9.68; N, 12.23, found: C, 73.52; H, 9.60; N, 12.33. *R_f_*: 0.75.

*N*-Dodecyl-5-(4-methoxyphenyl)-1,3,4-oxadiazol-2-amine **3c**. White solid, yield 90%; mp 116–117 °C. IR (ATR): 606, 634, 724, 739, 903, 828, 838, 956, 1029, 1049, 1173, 1266, 1299, 1469, 1506, 1587, 1614, 2850, 2921, 3266 cm^−1^. ^1^H NMR (600 MHz, DMSO-*D*_6_) δ 7.70 (2H, d, *J* = 8.4 Hz, H2, H6), 7.35–7.32 (1H, m, NH), 7.03 (2H, d, *J* = 8.4 Hz, H3, H5), 3.80 (3H, s, OCH_3_), 3.19 (2H, q, *J* = 6.6 Hz, NH-CH_2_), 1.55 (2H, p, *J* = 7.0 Hz, NH-CH_2_-CH_2_), 1.29–1.20 (18H, m, C^3^H_2_, C^4^H_2_, C^5^H_2_, C^6^H_2_, C^7^H_2_, C^8^H_2_, C^9^H_2_, C^10^H_2_, C^11^H_2_), 0.83 (3H, t, *J* = 6.7 Hz, CH_3_). ^13^C NMR (151 MHz, DMSO-*D*_6_) δ 164.03, 161.56, 158.01, 127.41, 117.72, 115.28, 55.99, 43.30, 31.74, 29.48, 29.46, 29.44, 29.43, 29.41, 29.14, 29.11, 26.71, 22.48, 14.27. Elemental analysis for C_21_H_33_N_3_O_2_ (359.51); calculated C, 70.16; H, 9.25; N, 11.69, found: C, 70.11; H, 9.15; N, 11.81. *R_f_*: 0.69.

5-[4-(*tert*-Butyl)phenyl]-*N*-dodecyl-1,3,4-oxadiazol-2-amine **3d**. White solid, yield 87%; mp 97–98 °C. IR (ATR): 614, 636, 725, 836, 852, 902, 1016, 1049, 1114, 1269, 1363, 1468, 1500, 1544, 1613, 2851, 2921, 3261 cm^−1^. ^1^H NMR (600 MHz, DMSO-*D*_6_) δ 7.70 (2H, d, *J* = 8.5 Hz, H3, H5), 7.50 (2H, d, *J* = 8.5 Hz, H2, H6), 7.41 (1H, s, NH), 3.20 (2H, td, *J* = 7.1, 5.7 Hz, NH-CH_2_), 1.56 (2H, p, *J* = 7.1 Hz, NH-CH_2_-CH_2_), 1.28 (9H, s, C(CH_3_)_3_), 1.25–1.20 (18H, m, C^3^H_2_, C^4^H_2_, C^5^H_2_, C^6^H_2_, C^7^H_2_, C^8^H_2_, C^9^H_2_, C^10^H_2_, C^11^H_2_), 0.82 (3H, t, *J* = 7.0 Hz, CH_3_). ^13^C NMR (151 MHz, DMSO-*D*_6_) δ 164.46, 157.36, 132.75, 127.53, 124.27, 124.12, 43.28, 31.74, 29.48, 29.44, 29.43, 29.41, 29.39, 29.37, 29.36, 29.34, 29.12, 26.68, 22.49, 14.27. *R_f_*: 0.77.

*N*-Dodecyl-5-(4-nitrophenyl)-1,3,4-oxadiazol-2-amine **3e**. White solid, yield 90%; mp 127–128 °C. IR (ATR): 680, 719, 733, 857, 953, 1013, 1047, 1072, 1296, 1350, 1479, 1535, 1507, 1562, 1605, 2849, 2916, 3250 cm^−1^. ^1^H NMR (600 MHz, DMSO-*D*_6_) δ 8.32–8.29 (2H, m, H2, H6), 8.01–7.99 (2H, m, H3, H5), 7.76 (1H, t, *J* = 5.8 Hz, NH), 3.25 (2H, q, *J* = 6.1 Hz, NH-CH_2_), 1.57 (2H, p, *J* = 7.1 Hz, NH-CH_2_-CH_2_), 1.26–1.19 (18H, m, C^3^H_2_, C^4^H_2_, C^5^H_2_, C^6^H_2_, C^7^H_2_, C^8^H_2_, C^9^H_2_, C^10^H_2_, C^11^H_2_), 0.82 (3H, t, *J* = 6.9 Hz, CH_3_). ^13^C NMR (151 MHz, DMSO-*D*_6_) δ 164.98, 156.77, 148.72, 130.52, 126.61, 124.97, 43.30, 31.74, 29.48, 29.46, 29.44, 29.43, 29.41, 29.33, 29.12, 26.68, 22.48, 14.25. Elemental analysis for C_20_H_30_N_4_O_3_ (374.49); calculated C, 64.15; H, 8.08; N, 14.96, found: C, 64.26; H, 8.00; N, 15.08. *R_f_*: 0.85.

5-[4-(Dimethylamino)phenyl]-*N*-dodecyl-1,3,4-oxadiazol-2-amine **3f**. White solid, yield 60%; mp 118–121 °C. IR (ATR): 612, 664, 721, 739, 815, 952, 1047, 1064, 1164, 1196, 1227, 1270, 1369, 1468, 1515, 1578, 1614, 1687, 2848, 2916, 3237 cm^−1^. ^1^H NMR (500 MHz, DMSO-*D*_6_) δ 7.77 (2H, d, *J* = 8.8 Hz, H3, H5), 7.59 (2H, d, *J* = 8.9 Hz, H2, H6), 6.80 (1H, t, *J* = 5.7 Hz, NH), 3.19 (2H, q, *J* = 6.6 Hz, NH-CH_2_), 2.97 (6H, s, N-CH_3_), 1.54 (2H, p, *J* = 7.1 Hz, NH-CH_2_-CH_2_), 1.33–1.16 (18H, m, C^3^H_2_, C^4^H_2_, C^5^H_2_, C^6^H_2_, C^7^H_2_, C^8^H_2_, C^9^H_2_, C^10^H_2_, C^11^H_2_), 0.84 (3H, t, *J* = 6.8 Hz, CH_3_). ^13^C NMR (126 MHz, DMSO-*D*_6_) δ 161.84, 160.94, 152.33, 127.63, 112.08, 111.90, 42.28, 31.49, 29.23, 29.19, 29.13, 29.05, 28.91, 28.70, 27.07, 26.12, 22.29, 14.15. Elemental analysis for C_22_H_36_N_4_O (372.56); calculated C, 70.93; H, 9.74; N, 15.04, found: C, 80.02; H, 9.81; N, 14.95. *R_f_*: 0.74.

*N*-Dodecyl-5-(4-fluorophenyl)-1,3,4-oxadiazol-2-amine **3g**. White solid, yield 81%; mp 104–105 °C. IR (ATR): 609, 660, 737, 820, 841, 1029, 1050, 1158, 1249, 1268, 1286, 1412, 1471, 1505, 1609, 1623, 2849, 2917, 2954, 3271 cm^−1^. ^1^H NMR (600 MHz, DMSO-*D*_6_) δ 7.83–7.79 (2H, m, H2, H6), 7.45 (1H, s, NH), 7.31–7.27 (2H, m, H3, H5), 3.21 (2H, td, *J* = 7.1, 5.7 Hz, NH-CH_2_), 1.55 (2H, p, *J* = 7.1 Hz, NH-CH_2_-CH_2_), 1.27–1.19 (18H, m, C^3^H_2_, C^4^H_2_, C^5^H_2_, C^6^H_2_, C^7^H_2_, C^8^H_2_, C^9^H_2_, C^10^H_2_, C^11^H_2_), 0.82 (3H, t, *J* = 6.9 Hz, CH_3_). ^13^C NMR (151 MHz, DMSO-*D*_6_) δ 164.47 (d, *J* = 32.7 Hz), 162.93, 157.32, 128.10 (d, *J* = 8.8 Hz), 121.78 (d, *J* = 3.2 Hz), 116.77 (d, *J* = 22.4 Hz), 43.29, 31.74, 29.48, 29.44, 29.43, 29.41, 29.37, 29.14, 29.11, 26.70, 22.48, 14.24. Elemental analysis for C_20_H_30_FN_3_O (347.48); calculated C, 69.13; H, 8.70; N, 12.09, found: C, 69.24; H, 8.69; N, 11.55. *R_f_*: 0.71.

5-(4-Chlorophenyl)-*N*-dodecyl-1,3,4-oxadiazol-2-amine **3h**. White solid, yield 92%; mp 120–121 °C. IR (ATR): 621, 719, 736, 827, 836, 955, 1014, 1051, 1074, 1099, 1287, 1406, 1487, 1521, 1607, 1621, 2849, 2918, 2953, 3246 cm^−1^. ^1^H NMR (600 MHz, DMSO-*D*_6_) δ 7.78–7.75 (2H, m, H2, H6), 7.55–7.51 (3H, m, H3, H5, NH), 3.21 (2H, td, *J* = 7.0, 5.7 Hz, NH-CH_2_), 1.59–1.52 (2H, m, NH-CH_2_-CH_2_), 1.25–1.19 (18H, m, C^3^H_2_, C^4^H_2_, C^5^H_2_, C^6^H_2_, C^7^H_2_, C^8^H_2_, C^9^H_2_, C^10^H_2_, C^11^H_2_), 0.82 (3H, t, *J* = 7.0 Hz, CH_3_). ^13^C NMR (151 MHz, DMSO-*D*_6_) δ 164.45, 157.27, 135.53, 129.81, 127.35, 123.93, 43.28, 31.74, 29.48, 29.44, 29.43, 29.41, 29.36, 29.13, 29.11, 26.69, 22.48, 14.26. Elemental analysis for C_20_H_30_ClN_3_O (363.93); calculated C, 66.01; H, 8.31; N, 11.55, found: C, 66.12; H, 8.30; N, 11.67. *R_f_*: 0.75.

5-(4-Bromophenyl)-*N*-dodecyl-1,3,4-oxadiazol-2-amine **3i**. White solid, yield 96%; mp 129–130 °C. IR (ATR): 620, 720, 832, 1011, 1030, 1050, 1070, 1156, 1269, 1287, 1402, 1471, 1483, 1600, 1618, 2848, 2917, 2951, 3253 cm^−1^. ^1^H NMR (600 MHz, DMSO-*D*_6_) δ 7.71–7.66 (4H, m, H2, H3, H5, H6), 7.54–7.51 (1H, m, NH), 3.21 (2H, td, *J* = 7.0, 5.7 Hz, NH-CH_2_), 1.55 (2H, p, *J* = 7.1 Hz, NH-CH_2_-CH_2_), 1.29–1.19 (18H, m, C^3^H_2_, C^4^H_2_, C^5^H_2_, C^6^H_2_, C^7^H_2_, C^8^H_2_, C^9^H_2_, C^10^H_2_, C^11^H_2_), 0.82 (3H, t, *J* = 6.9 Hz, CH_3_). ^13^C NMR (151 MHz, DMSO-*D*_6_) δ 164.46, 157.36, 132.75, 127.53, 124.27, 124.12, 43.28, 31.74, 29.48, 29.46, 29.44, 29.43, 29.41, 29.36, 29.12, 26.68, 22.49, 14.27. Elemental analysis for C_20_H_30_BrN_3_O (408.38); calculated C, 58.82; H, 7.40; N, 10.29, found: C, 58.97; H, 7.49; N, 10.37. *R_f_*: 0.75.

*N*-Dodecyl-5-(4-iodophenyl)-1,3,4-oxadiazol-2-amine **3j**. White solid, yield 74%; mp 150–152 °C. IR (ATR): 625, 711, 720, 736, 829, 1007, 1049, 1062, 1156, 1269, 1287, 1339, 1472, 1480, 1524, 1598, 1621, 2849, 2920, 2955, 3240 cm^−1^. ^1^H NMR (600 MHz, DMSO-*D*_6_) δ 7.87–7.84 (2H, m, H3, H5), 7.55–7.51 (3H, m, H2, H6, NH), 3.21 (2H, q, *J* = 6.6 Hz, NH-CH_2_), 1.55 (2H, p, *J* = 7.1 Hz, NH-CH_2_-CH_2_), 1.24–1.20 (18H, m, C^3^H_2_, C^4^H_2_, C^5^H_2_, C^6^H_2_, C^7^H_2_, C^8^H_2_, C^9^H_2_, C^10^H_2_, C^11^H_2_), 0.82 (3H, t, *J* = 7.0 Hz, CH_3_). ^13^C NMR (151 MHz, DMSO-*D*_6_) δ 164.44, 157.53, 138.60, 127.39, 124.56, 97.15, 43.28, 31.74, 29.47, 29.46, 29.44, 29.42, 29.40, 29.35, 29.11, 26.68, 22.49, 14.28. Elemental analysis for C_20_H_30_IN_3_O (455.38); calculated C, 52.75; H, 6.64; N, 9.23, found: C, 52.89; H, 6.71; N, 9.17. *R_f_*: 0.77.

5-(3,5-Dinitrophenyl)-*N*-dodecyl-1,3,4-oxadiazol-2-amine **3k**. Yellow solid, yield 92%; mp 131–133 °C. IR (ATR): 685, 728, 911, 1027, 1072, 1135, 1343, 1352, 1469, 1545, 1558, 1568, 1671, 2850, 2921, 3100 cm^−1^. ^1^H NMR (500 MHz, DMSO-*D*_6_) δ 8.86 (2H, d, *J* = 2.1 Hz, H2, H6), 8.76 (1H, t, *J* = 2.1 Hz, H4), 8.13 (1H, t, *J* = 5.7 Hz, NH-CH_2_), 3.27 (2H, q, *J* = 6.7 Hz, NH-CH_2_), 1.58 (2H, p, *J* = 7.1 Hz, NH-CH_2_-CH_2_), 1.37–1.20 (18H, m, C^3^H_2_, C^4^H_2_, C^5^H_2_, C^6^H_2_, C^7^H_2_, C^8^H_2_, C^9^H_2_, C^10^H_2_, C^11^H_2_), 0.85 (3H, t, *J* = 6.8 Hz, CH_3_). ^13^C NMR (126 MHz, DMSO-*D*_6_) δ 164.47, 154.91, 148.89, 127.01, 124.66, 119.33, 42.75, 31.48, 29.23, 29.20, 29.17, 29.16, 28.90, 28.87, 28.85, 26.33, 22.28, 14.12. Elemental analysis for C_20_H_29_N_5_O_5_ (419.48); calculated C, 57.27; H, 6.97; N, 16.70, found: C, 57.34; H, 7.00; N, 16.78. *R_f_*: 0.62.

*N*-Dodecyl-5-(pyridin-4-yl)-1,3,4-oxadiazol-2-amine **3l**. The synthesis and characterization of the compound was published previously by our group [21].

*N*-Dodecyl-5-(pyridin-3-yl)-1,3,4-oxadiazol-2-amine **3m**. White solid, yield 87%; mp 121–122 °C. IR (ATR): 625, 706, 718, 733, 820, 961, 1016, 1056, 1079, 1390, 1430, 1471, 1479, 1551, 1619, 2851, 2917, 2953, 3269 cm^−1^. ^1^H NMR (600 MHz, DMSO-*D*_6_) δ 8.95 (1H, d, *J* = 2.2 Hz, H2), 8.64 (1H, dd, *J* = 4.8, 1.6 Hz, H6), 8.11 (1H, dt, *J* = 8.0, 2.0 Hz, H4), 7.62–7.58 (1H, m, NH), 7.50 (1H, ddd, *J* = 8.0, 4.8, 0.9 Hz, H5), 3.23 (2H, td, *J* = 7.0, 5.7 Hz, NH-CH_2_), 1.56 (2H, p, *J* = 7.1 Hz, NH-CH_2_-CH_2_), 1.27–1.20 (18H, m, C^3^H_2_, C^4^H_2_, C^5^H_2_, C^6^H_2_, C^7^H_2_, C^8^H_2_, C^9^H_2_, C^10^H_2_, C^11^H_2_), 0.82 (3H, t, *J* = 6.9 Hz, CH_3_). ^13^C NMR (151 MHz, DMSO-*D*_6_) δ 164.64, 156.07, 151.45, 146.49, 132.99, 124.58, 121.46, 43.31, 31.74, 29.47, 29.45, 29.43, 29.40, 29.35, 29.13, 29.10, 26.69, 22.48, 14.26. Elemental analysis for C_19_H_30_N_4_O (330.48); calculated C, 69.05; H, 9.15; N, 16.95, found: C, 69.14; H, 9.14; N, 17.08. *R_f_*: 0.47.

*N*-Dodecyl-5-(pyridin-2-yl)-1,3,4-oxadiazol-2-amine **3n**. White solid, yield 47%; mp 114–116 °C. IR (ATR): 622, 685, 717, 741, 786, 961,1096, 1149, 1288, 1392, 1439, 1460, 1479, 1559, 1591, 1632, 2852, 2918, 3266 cm^−1^. ^1^H NMR (500 MHz, DMSO-*D*_6_) δ 10.37 (1H, s, NH-CH_2_), 8.65 (1H, dd, *J* = 4.4, 1.3 Hz, H6), 7.98–7.91 (2H, m, H3, H4), 7.49 (1H, ddd, *J* = 6.8, 4.6, 1.8 Hz, H5), 3.23 (2H, q, *J* = 6.7 Hz, NH-CH_2_), 1.56 (2H, p, *J* = 7.0 Hz, NH-CH_2_-CH_2_), 1.33–1.17 (18H, m, C^3^H_2_, C^4^H_2_, C^5^H_2_, C^6^H_2_, C^7^H_2_, C^8^H_2_, C^9^H_2_, C^10^H_2_, C^11^H_2_), 0.83 (3H, t, *J* = 6.8 Hz, CH_3_). ^13^C NMR (126 MHz, DMSO-*D*_6_) δ 164.36, 157.51, 150.01, 143.71, 137.64, 125.08, 121.35, 45.50, 42.70, 31.46, 29.21, 29.17, 29.13, 28.87, 28.82, 26.32, 22.25, 14.11, 8.59. Elemental analysis for C_19_H_30_N_4_O (330.48); calculated C, 69.05; H, 9.15; N, 16.95, found: C, 69.15; H, 9.26; N, 16.81. *R_f_*: 0.75.

*N*-Dodecyl-5-(pyrimidin-4-yl)-1,3,4-oxadiazol-2-amine **3o**. Pinkish solid, yield 76%; mp 154–155 °C. IR (ATR): 665, 683, 723, 845, 971, 989, 1060, 1074, 1156, 1292, 1397, 1470, 1481, 1582, 1625, 2850, 2916, 2954, 3032, 3269 cm^−1^. ^1^H NMR (500 MHz, DMSO-*D*_6_) δ 9.26 (1H, s, H2), 8.93 (1H, d, *J* = 5.2 Hz, H5), 8.03 (1H, s, NH-CH_2_), 7.96 (1H, d, *J* = 5.2 Hz, H6), 3.28 (2H, t, *J* = 7.0 Hz, NH-CH_2_), 1.60 (2H, p, *J* = 7.0 Hz, NH-CH_2_-CH_2_), 1.38–1.20 (18H, m, C^3^H_2_, C^4^H_2_, C^5^H_2_, C^6^H_2_, C^7^H_2_, C^8^H_2_, C^9^H_2_, C^10^H_2_, C^11^H_2_), 0.85 (3H, t, *J* = 6.7 Hz, CH_3_). ^13^C NMR (126 MHz, DMSO-*D*_6_) δ 164.71, 158.76, 158.19, 155.89, 149.85, 117.16, 42.54, 31.05, 28.77, 28.74, 28.73, 28.71, 28.70, 28.57, 28.41, 25.95, 21.80, 13.60. Elemental analysis for C_18_H_29_N_5_O (331.46); calculated C, 65.23; H, 8.82; N, 21.13, found: C, 65.31; H, 8.90; N, 21.11. *R_f_*: 0.58.

*N*-Dodecyl-5-(pyrazin-2-yl)-1,3,4-oxadiazol-2-amine **3p**. White solid, yield 61%; mp 130–131 °C. IR (ATR): 716, 737, 756, 850, 967, 1015, 1041, 1058, 1098, 1175, 1288, 1389, 1423, 1472, 1523, 1627, 2852, 2918, 3294 cm^−1^. ^1^H NMR (500 MHz, DMSO-*D*_6_) δ 9.16 (1H, d, *J* = 1.5 Hz, H3), 8.73–8.70 (2H, m, H5, H6), 7.87 (1H, s, NH-CH_2_), 3.28 (2H, q, *J* = 6.6 Hz, NH-CH_2_), 1.60 (2H, p, *J* = 7.0 Hz, NH-CH_2_-CH_2_), 1.38–1.20 (18H, m, C^3^H_2_, C^4^H_2_, C^5^H_2_, C^6^H_2_, C^7^H_2_, C^8^H_2_, C^9^H_2_, C^10^H_2_, C^11^H_2_), 0.85 (3H, t, *J* = 6.8 Hz, CH_3_). ^13^C NMR (126 MHz, DMSO-*D*_6_) δ 164.45, 155.48, 145.02, 144.33, 142.09, 139.64, 42.58, 31.04, 28.77, 28.74, 28.72, 28.70, 28.68, 28.59, 28.41, 25.96, 21.79, 13.58. Elemental analysis for C_18_H_29_N_5_O (331.46); calculated C, 65.23; H, 8.82; N, 21.13, found: C, 65.20; H, 8.74; N, 21.00. *R_f_*: 0.60.

5-(2-Bromopyridin-4-yl)-*N*-dodecyl-1,3,4-oxadiazol-2-amine **3q**. White solid, yield 50%; mp 92–93 °C. IR (ATR): 672, 689, 724, 736, 747, 850, 872, 1005, 1025, 1038, 1075, 1089, 1130, 1302, 1367, 1461, 1495, 1566, 1596, 1657, 2850, 2919, 2954, 3082, 3190 cm^−1^. ^1^H NMR (500 MHz, DMSO-*D*_6_) δ 8.52 (1H, d, *J* = 5.1 Hz, H6), 8.11 (1H, t, *J* = 5.7 Hz, NH), 7.86 (1H, d, *J* = 1.4 Hz, H3), 7.75 (1H, dd, *J* = 5.2, 1.5 Hz, H5), 3.25 (2H, q, *J* = 6.6 Hz, NH-CH_2_), 1.56 (2H, p, *J* = 6.9 Hz, NH-CH_2_-CH_2_), 1.33–1.16 (18H, m, C^3^H_2_, C^4^H_2_, C^5^H_2_, C^6^H_2_, C^7^H_2_, C^8^H_2_, C^9^H_2_, C^10^H_2_, C^11^H_2_), 0.83 (3H, t, *J* = 6.7 Hz, CH_3_). ^13^C NMR (126 MHz, DMSO-*D*_6_) δ 164.52, 154.84, 151.76, 142.28, 134.17, 122.64, 118.59, 42.71, 31.47, 29.21, 29.18, 29.13, 29.12, 28.88, 28.86, 28.79, 26.28, 22.26, 14.12. Elemental analysis for C_19_H_29_BrN_4_O (409.37); calculated C, 55.75; H, 7.14; N, 13.69, found: C, 55.89; H, 7.22; N, 13.80. *R_f_*: 0.78.

*N*-Dodecyl-5-(pyridazin-4-yl)-1,3,4-oxadiazol-2-amine **3r**. Brown solid, yield 41%; mp 142–143 °C. IR (ATR): 665, 719, 727, 882, 975, 1058, 1340, 1380, 1395, 1471, 1480, 1527, 1568, 1617, 1662, 2850, 2917, 2953, 3267 cm^−1^. ^1^H NMR (500 MHz, DMSO-*D*_6_) δ 9.55 (1H, dd, *J* = 2.3, 1.3 Hz, H3), 9.37 (1H, dd, *J* = 5.5, 1.3 Hz, H6), 8.19 (1H, t, *J* = 5.7 Hz, NH-CH_2_), 7.91 (1H, dd, *J* = 5.4, 2.3 Hz, H5), 3.26 (2H, q, *J* = 6.6 Hz, NH-CH_2_), 1.56 (2H, p, *J* = 7.0 Hz, NH-CH_2_-CH_2_), 1.34–1.20 (18H, m, C^3^H_2_, C^4^H_2_, C^5^H_2_, C^6^H_2_, C^7^H_2_, C^8^H_2_, C^9^H_2_, C^10^H_2_, C^11^H_2_), 0.83 (3H, t, *J* = 6.7 Hz, CH_3_). ^13^C NMR (126 MHz, DMSO-*D*_6_) δ 164.67, 154.11, 152.28, 146.46, 122.72, 121.04, 42.75, 31.46, 29.20, 29.17, 29.13, 29.10, 28.87, 28.84, 28.81, 26.30, 22.25, 14.10. Elemental analysis for C_18_H_29_N_5_O (331.46); calculated C, 65.23; H, 8.82; N, 21.13, found: C, 65.31; H, 8.91; N, 21.24. *R_f_*: 0.42.

5-(Pyridin-4-yl)-1,3,4-oxadiazol-2-thiol **2s** (2 mmol, 358 mg) was suspended together with 2 equivalents of potassium carbonate (4 mmol, 552 mg) in 30 mL of *N*,*N*-dimethylformamide. Then, 1-bromododecane was added in one portion (2 mmol, 498 mg). Reaction was allowed to react for 5 h. The reaction mixture was evaporated to dryness on a rotary evaporator and transferred to a separatory funnel with 30 mL of EtOAc and 30 mL of demineralized water. The organic phase was washed with 30 mL of a saturated brine. The organic phase was dried over anhydrous sodium sulfate for 45 min, then the desiccant was separated, and the mixture was evaporated to dryness. The crude product was overlaid with dry DCM and stored at −20 °C. The solid product was filtered off and washed twice with 5 mL of ice-cold DCM. 

2-(Dodecylthio)-5-(pyridin-4-yl)-1,3,4-oxadiazole **3s**. Yellow solid, yield 100%; mp 63–64 °C. IR (ATR): 707, 723, 835, 961, 989, 1063, 1083, 1182, 1226, 1415, 1460, 1539, 1608, 2850, 2919, 2955 cm^−1^. ^1^H NMR (500 MHz, DMSO-*D*_6_) δ 8.82–8.79 (2H, m, H2, H6), 7.90–7.87 (2H, m, H3, H5), 3.32 (2H, t, *J* = 7.2 Hz, S-CH_2_), 1.76 (2H, p, *J* = 7.4 Hz, S-CH_2_-CH_2_), 1.38 (2H, p, *J* = 7.3 Hz, C^3^H_2_), 1.31–1.16 (16H, m, C^4^H_2_, C^5^H_2_, C^6^H_2_, C^7^H_2_, C^8^H_2_, C^9^H_2_, C^10^H_2_, C^11^H_2_), 0.83 (3H, t, *J* = 6.8 Hz, CH_3_). ^13^C NMR (126 MHz, DMSO-*D*_6_) δ 165.63, 163.72, 151.13, 130.33, 120.14, 32.28, 31.48, 29.21, 29.19, 29.12, 29.08, 29.03, 28.89, 28.55, 27.96, 22.28, 14.14. Elemental analysis for C_19_H_29_N_3_OS (347.52); calculated C, 65.67; H, 8.41; N, 12.09, found: C, 65.79; H, 8.52; N, 12.09. *R_f_*: 0.72.

*N*-Dodecyl-5-(pyridin-4-yl)-1,3,4-thiadiazol-2-amine **3t**. Yellow solid, yield 68%; mp 152–154 °C. IR (ATR): 632, 683, 719, 802, 816, 992, 1010, 1035, 1121, 1195, 1375, 1420, 1468, 1494, 1523, 1549, 1592, 1635, 2854, 2920, 2955, 3194 cm^−1^. ^1^H NMR (500 MHz, DMSO-*D*_6_) δ 8.76 (2H, d, *J* = 6.1 Hz, H2, H6), 8.01 (2H, d, *J* = 6.0 Hz, H3), 3.40 (2H, t, *J* = 7.0 Hz, NH-CH_2_), 1.65 (2H, p, *J* = 7.2 Hz, NH-CH_2_-CH_2_), 1.41–1.21 (18H, m, C^3^H_2_, C^4^H_2_, C^5^H_2_, C^6^H_2_, C^7^H_2_, C^8^H_2_, C^9^H_2_, C^10^H_2_, C^11^H_2_), 0.86 (3H, t, *J* = 6.7 Hz, CH_3_). ^13^C NMR (126 MHz, DMSO-*D*_6_) δ 170.82, 159.97, 145.49, 142.65, 121.18, 45.14, 30.86, 28.59, 28.56, 28.54, 28.52, 28.26, 28.22, 28.12, 25.97, 21.58, 13.34. Elemental analysis for C_19_H_30_N_4_S (346.54); calculated C, 65.85; H, 8.73; N, 16.17, found: C, 65.99; H, 8.70; N, 16.25. *R_f_*: 0.45.

*N*′-Tridecylideneisonicotinohydrazide **2u** (0.5 mmol, 159 mg) was dissolved in 30 mL of DMSO, iodine (1.2 eq., 0.6 mmol, 152 mg) and potassium carbonate (3 eq., 1.5 mmol, 207 mg) was added to the solution, and heated to 100 °C. After 3 h, the mixture was evaporated to dryness on a rotary evaporator and transferred to a separatory funnel with 30 mL of EtOAc acetate and 30 mL of demineralized water. The organic phase was washed with 30 mL of a saturated Na_2_S_2_O_3_ solution and finally 30 mL of a saturated brine. The organic phase was dried over anhydrous sodium sulfate for 45 min, then the desiccant was separated, and the mixture was evaporated to dryness. The crude product was overlaid with dry DCM and stored at −20 °C. The solid product was filtered off on and washed twice with 5 mL of ice-cold DCM [36].

2-Dodecyl-5-(pyridin-4-yl)-1,3,4-oxadiazole **3u**. Yellow solid, yield 76%; mp 75–76 °C. IR (ATR): 636, 704, 724, 730, 833, 989, 1023, 1099, 1182, 1232, 1417, 1464, 1473, 1541, 1565, 1608, 2849, 2872, 2917, 2955 cm^−1^. ^1^H NMR (500 MHz, DMSO-*D*_6_) δ 8.82–8.80 (2H, m, H2, H6), 7.90–7.88 (2H, m, H3, H5), 2.95 (2H, t, *J* = 7.4 Hz, C^1^H_2_), 1.78 (2H, p, *J* = 7.4 Hz, C^2^H_2_), 1.38 (2H, p, *J* = 7.3 Hz, C^3^H_2_), 1.34–1.20 (16H, m, C^4^H_2_, C^5^H_2_, C^6^H_2_, C^7^H_2_, C^8^H_2_, C^9^H_2_, C^10^H_2_, C^11^H_2_), 0.85 (3H, t, *J* = 6.8 Hz, CH_3_). ^13^C NMR (126 MHz, DMSO-*D*_6_) δ 167.84, 162.32, 150.84, 130.58, 119.96, 31.15, 28.86, 28.85, 28.80, 28.66, 28.52, 28.35, 28.15, 25.63, 24.54, 21.92, 13.72. Elemental analysis for C_19_H_29_N_3_O (315.46); calculated C, 72.34; H, 9.27; N, 13.32, found: C, 72.41; H, 9.30; N, 13.22. *R_f_*: 0.64.

### 3.2. Acetyl- and Butyrylcholinesterase Inhibition

Enzyme inhibition activity of **3** quantified as IC_50_ values was determined spectrophotometrically using modified Ellman’s method [38]. It has widely been used for in vitro screening of potential AChE and BChE inhibitors. The enzyme activity is measured indirectly by determination of 5-mercapto-2-nitrobenzoic acid ion formed after reaction of the thiol reagent 5,5′-disulfanediyl-bis(2-nitrobenzoic acid) (DTNB) and thiocholine, a product of acetylthiocholine (ATCh) and butyrylthiocholine (BTCh) as substrates for hydrolysis by AChE and BChE, respectively [39].

The enzyme activity in final reaction mixture (2000 µL) was 0.2 U/mL, concentration of ATCh (or BTCh) 40 µM and concentration of DTNB 100 µM for all reactions. The compounds **3** were dissolved in DMSO and then diluted in demineralized water (conductivity 3 μS, equipment supplier BKG Water Treatment, Hradec Králové, Czech Republic) to the concentration of 1000 µM. For all the tested compounds and rivastigmine, five different concentrations of inhibitor in final reaction mixture were used. The final concentration of DMSO was 0.2%. All experiments were carried in triplicates. The average values of reaction rate (v_0_-uninhibited reaction, v_i_-inhibited reaction) were used for construction of the dependence v_0_/v_i_ vs. concentration of inhibitor. From obtained equation of regression curve, IC_50_ values were calculated.

Then, we assessed reversibility of AChE inhibition caused by the most potent inhibitor **3t**. Three different inhibitor concentrations (1, 10, and 100 µM) were chosen. The aim was to observe the effect of the inhibitor on enzyme activity (A) over time. Based on this, it is possible to distinguish reversible and irreversible inhibition. The procedure was analogous to the determination of enzyme activity. The reaction mixture containing phosphate buffer, AChE and **3t** (at one of the chosen concentrations) was prepared and stirred vigorously. At given times (5, 10, 15, 20, 30, 40, 50, 60, 260, and 1450 min), DTNB and ATCh were added to the sample taken from the reaction mixture, mixed rapidly, and the absorbance was measured to quantify the enzyme activity. Based on knowledge of the enzyme activity in the absence of the inhibitor (i.e., 100% activity), the percentages of residual enzyme activity in presence of inhibitor were calculated. Then, the dependence of percentage of residual enzyme activity (% A) on time was constructed. Based on these kinetic data, it is possible to distinguish between reversible, pseudo-irreversible and irreversible inhibition.

For the determination of the type of inhibition of the most active inhibitor **3t**, Lineweaver–Burk plot [34] was used. The procedure was analogous to the determination of IC_50_. The enzyme activity in the final reaction mixture (2000 µL) was 0.2 U/mL, concentration of ATCh 20–80 µM and concentration of DTNB was 100 µM. For each of the substrate concentrations, four different concentrations of the inhibitor were used (10, 25, 35 and 50 µM). The dependence of absorbance vs. time was observed and the reaction rate was calculated. All experiments were carried in duplicates, and the average values of reaction rate were used for the construction of the Lineweaver–Burk plot. From obtained equations of regression curves in Lineweaver–Burk plot, the values of Michaelis constant (K_M_) and maximum velocity (V_m_) were calculated. Based on these results, the type of inhibition was identified.

Acetylcholinesterase was used from electric eel (*Electrophorus electricus* L.; *Ee*AChE) and butyrylcholinesterase originated from equine serum (EqBChE). A drug rivastigmine was used as a reference dual enzyme inhibitor. All the enzymes, substrates and rivastigmine were purchased from Merck (Prague, Czech Republic).

### 3.3. Molecular Docking

Crystallographic structures of human AChE and human BChE were obtained from protein data bank (www.rcsb.org accessed on 30 January 2022; pdb codes 4PQE and 1POI, respectively). The 3D structures of ligands were prepared in Chem3D Pro 19.1 (ChemBioOffice 2019 Package, CambridgeSoft, Cambridge, MA, USA). In the preparation process, water molecules were removed from the enzymes and the structures of enzymes and ligands were optimized using UCSF Chimera software package (Amber force field) [40]. Docking was performed using Autodock Vina [41] and Autodock 4.2 [42]; a Lamarckian genetic algorithm was used. The 3D affinity grid box was designed to include the full active and peripheral site of AChE and BChE. The number of grid points in the X-, Y- and Z-axes was 20, 20 and 20, respectively, with grid points separated by 1 Å (Autodock Vina), and 40, 40 and 40, respectively, with grid points separated by 0.4 Å (Autodock 4). The graphic visualizations of the ligand-enzyme interactions were prepared in PyMOL (The PyMOL Molecular Graphics System, Version 1.5 Schrödinger, LLC, New York, NY, USA).

## 4. Conclusions

A series of twenty 1,3,4-oxadiazoles and one 1,3,4-thiadiazole were designed, prepared, and evaluated as potential inhibitors of acetylcholinesterase and butyrylcholinesterase. Almost all of them showed significant inhibition of both cholinesterases with IC_50_ values in the micromolar range. Their activity toward AChE was found to be comparatively higher than that toward BChE. Many of them showed higher inhibitory effect against AChE than the drug rivastigmine. We described structure–activity relationships. The most active compound, thiadiazole decorated with 4-pyridyl and dodecylamine, was the best inhibitor of both enzymes. It represents a reversible, mixed-type AchE inhibitor. The interactions of the most potent inhibitors with target structures were also investigated using molecular docking, which showed that the binding is different for particular acetylcholine hydrolysing enzymes.

Other types of biological activities, especially antimicrobial, will be investigated in the future.

## Figures and Tables

**Table 1:** Inhibition of AChE and BChE and selectivity indexes of the heterocycles **3**.

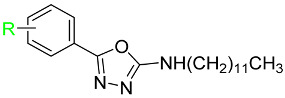					
**Code**	**R**	**IC_50_ AChE (µM)**	**IC_50_ BChE (µM)**	**BChE/AChE Ratio**	**Log *P_o/w_***
**3a**	H	40.11 ± 0.54	149.47 ± 1.69	3.7	4.34
**3b**	4-CH_3_	**34.65 ± 0.96**	**89.97 ± 2.35**	2.6	4.57
**3c**	4-OCH_3_	**33.86 ± 0.27**	224.53 ± 10.96	6.6	4.00
**3d**	4-*t*-Bu	65.24 ± 0.72	151.92 ± 6.02	2.3	5.21
**3e**	4-NO_2_	70.46 ± 0.59	105.16 ± 0.94	1.5	3.30
**3f**	4-N(CH_3_)_2_	87.99 ± 0.81	316.48 ± 6.22	3.6	4.22
**3g**	4-F	**36.73 ± 1.59**	266.68 ± 2.59	7.3	4.72
**3h**	4-Cl	50.23 ± 1.88	443.68 ± 8.50	8.8	4.83
**3i**	4-Br	39.99 ± 0.98	369.29 ± 5.82	9.2	4.94
**3j**	4-I	**37.83 ± 1.83**	481.94 ± 6.15	**12.7**	5.05
**3k**	3,5-di-NO_2_	40.98 ± 0.11	384.43 ± 5.34	9.4	2.36
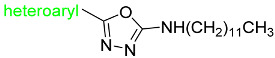					
**3l**	4-pyridyl	50.22 ± 0.79	223.03 ± 1.54	4.4	3.29
**3m**	3-pyridyl	64.21 ± 0.93	369.31 ± 3.83	5.8	3.29
**3n**	2-pyridyl	60.49 ± 2.42	315.43 ± 1.96	5.2	3.29
**3o**	4-pyrimidinyl	45.52 ± 0.75	298.34 ± 2.19	6.6	2.64
**3p**	2-pyrazinyl	92.13 ± 6.11	451.70 ± 59.26	4.9	2.24
**3q**	2-Br-4-pyridyl	73.37 ± 2.06	224.24 ± 1.29	3.1	3.89
**3r**	4-pyridazinyl	81.46 ± 4.14	237.83 ± 13.84	2.9	3.05
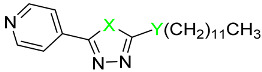					
**3s**	X=O, Y=S	91.56 ± 7.14	>500	>5.5	3.71
**3t**	X=S, Y=NH	**12.78 ± 0.03**	**53.05 ± 3.68**	4.2	3.30
**3u**	X=O, Y is missing	99.18 ± 3.26	288.98 ± 1.55	2.9	3.44
Rivastigmine		56.10 ± 1.41	38.40 ± 1.97	1.46	-

IC_50_ values are expressed as the mean ± SD (*n* = three independent experiments). The lowest IC_50_ values for each enzyme are given in bold as well as the most selective AChE inhibitor.

## Data Availability

Data is contained within the article.
